# Unlocking Modulation Rule of Heterointerface Engineering Induced D‐Band Center on Polysulfides Conversion in Lithium–Sulfur Batteries

**DOI:** 10.1002/advs.202501940

**Published:** 2025-03-06

**Authors:** Wenbin Li, Ni Wang, Jingjie Pei, Dongyan Li, Guiqiang Cao, Ruixian Duan, Jingjing Wang, Xifei Li

**Affiliations:** ^1^ Institute of Advanced Electrochemical Energy and School of Materials Science and Engineering Xi'an University of Technology Xi'an 710048 China; ^2^ Shaanxi Engineering Research Center of Key Materials for Lithium/Sodium‐ion Batteries Shaanxi International Joint Research Center of Surface Technology for Energy Storage Materials Key Laboratory of Advanced Batteries Materials for Electric Vehicles of China Petroleum and Chemical Industry Federation Xi'an 710048 China; ^3^ Xi'an North Huian Chemical Industries Company Xi'an Shaanxi 710302 China; ^4^ Guangdong Yuanneng Technologies Co Ltd Foshan Guangdong 528223 China

**Keywords:** d‐band center, heterointerface engineering, lithium–sulfur batteries, polysulfides conversion, sulfur cathode host

## Abstract

MOF‐derived heterostructures have been widely utilized as S hosts to address the three issues faced by the sulfur (S) cathode in lithium–sulfur batteries. Precisely pinpointing the active center in the heterostructure and unlocking the modulation rule of heterostructure on polysulfides (LiPSs) conversion are particularly important. Herein, the two homologous hetero‐hosts of ZIF‐67‐derived CoSe_2_@CoSe_0.25_S_1.75_/NC and CoSe_2_@CoS_2_/NC nanoparticles anchored in the nitrogen‐doped carbon (NC) matrix are successfully constructed, and confirm that Co at the heterointerface is the primary active center, rather than Co within the individual phases. It is revealed that the *ɛ*_d_ (d‐band center) of Co in the heterostructure can be a significant descriptor for the adsorption ability and conversion ability to LiPSs, and the discharge‐specific capacities, which describe a volcano curve as a function of *ε*_d_. It is also found that the best Li^+^ storage property appears at CoSe_2_@CoSe_0.25_S_1.75_ heterointerface among the five structures (CoSe_2_@CoSe_0.25_S_1.75_, CoSe_2_@CoS_2_, CoSe_0.25_S_1.75_, CoSe_2_, CoS_2_). The capacity is up to 600 mAh g^−1^ with a large retention rate of 73.3% at 0.2 C after 100 cycles, and the capacity decay rate is only 0.083% per cycle at 1 C during 300 long‐cycling process.

## Graphical abstract

The homologous heterostructure of ZIF‐67‐derived CoSe_2_@CoSe_0.25_S_1.75_ with a behavior of semi‐coherent phase boundary is constructed to modulate the d‐band center (*ε*_d_) of Co, which can be a significant descriptor for Li^+^ storage of S cathode. It is revealed that the adsorption and conversion ability to LiPSs and the discharge‐specific capacity describe a volcano curve as a function of *ɛ*_d_.

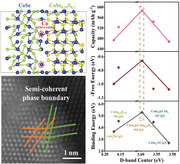

## 1 Introduction

In the context of “emissions peak and carbon neutrality” grand strategy, lithium‐ion batteries (LIBs) for electrochemical energy storage have become the focus of global development, especially in the field of new energy vehicles.^[^ ^1^ ^]^ With the acceleration of the electrification era, countries around the world are already developing electric aircraft and the corresponding national development strategies have been formulated. In China, the low‐altitude economy has been included in the 2024 government work report. The electric aircraft puts forward higher requirements for the energy density and safety of batteries, and the traditional LIBs can no longer meet these requirements. In this regard, the lithium–sulfur batteries (LSBs) with the low‐cost sulfur (S, theoretical specific capacity: 1675 mAh g^−1^) as cathode and the metal Li (theoretical specific capacity: 3860 mAh g^−1^) as anode, have been regarded as the potential power source of electric aircraft owing to its high energy density (2600 Wh kg^−1^).^[^ ^2^ ^]^ However, the S cathodes of LSBs are still constrained the poor conductivity of S and the discharge product Li_2_S/Li_2_S_2_, the severe volumetric respiration of S material, and the “shuttle effect” of polysulfide (LiPSs, initial discharge product).^[^ ^3^ ^]^

The metal‐organic framework (MOF) derivatives have been utilized as S hosts to address the above three issues, where the N‐doped carbon (NC) substrates contribute to improving the electrical conductivity, and the porous frameworks can well confine the severe volumetric respiration.^[^ ^4^ ^]^ Although the polar MOF derivatives are capable of chemically anchoring LiPSs and thus restraining their shuttle, the reversible conversion between liquid LiPSs and solid Li_2_S/Li_2_S_2_ still faces great challenges. As a result, extensive research has focused on MOF‐derived heterostructures, and two main views have been proposed.^[^ ^5^ ^]^ A mainstream view holds that heterostructures can realize the tandem catalytic process, where the two phases and the heterointerfaces are the active centers with different functions. Concretely, the phase with strong adsorption ability can chemically capture LiPSs and thus restrain the loss of active species, the phase with strong catalytic ability can promote the conversion reaction from liquid LiPSs to solid Li_2_S/Li_2_S_2_, and the heterointerface can induce the fast e^−^/LiPSs directional transformation between the two phases by the as‐formed interfacial built‐in electric field.^[^ ^6^ ^]^ These synergistic tandem effects of “adsorption‐transfer‐conversion” or “adsorption‐conversion” toward LiPSs contribute to well address the challenge of “shuttle effect.”^[^ ^7^ ^]^ For example, MOF‐derived Co/CoV_2_O_6_,^[^ ^8^ ^]^ V_8_C_7_/V_2_O_3_,^[^ ^9^ ^]^ and Co_9_S_8_/CoO^[^ ^10^ ^]^ Mo_2_N/SnO_2_^[^ ^11^ ^]^ heterostructures have been reported, where the former and latter are catalytic and adsorptive phases, respectively.

Another popular view is that the heterointerfaces as the active centers of LiPSs adsorption and conversion can directly restrain the loss of active species, and accelerate the reversible LiPS conversion, eventually addressing the challenge of “shuttle effect.”^[^ ^12^ ^]^ For example, the built‐in electric fields is constructed,^[^ ^13^ ^]^ the d‐band center of Co is downshifted,^[^ ^14^ ^]^ the spin splitting of Ni 3d orbital is converted from low to high spin,^[^ ^15^ ^]^ the charge redistribution and lattice distortion are realized,^[^ ^16^ ^]^ and the strong electron transfer behavior is modulated^[^ ^17^ ^]^ by designing ZnTe‐ZnO, Co/CoS_2_, NiS_2_/NiSe_2_, CoSe‐ZnSe and Cu_3_P‐Cu_2_O heterointerfaces, respectively. In addition, Zhu et al. enhanced the interaction between LiPSs and CoSe and thus accelerated the catalytic conversion of LiPSs by constructing the Co/CoSe junction.^[^ ^18^ ^]^ Wang et al. found that in the Mo_2_C/MoC heterostructure, the heterointerface and Mo_2_C/MoC contributed to long‐chain and short‐chain LiPSs conversion, respectively.^[^ ^19^ ^]^

Through the above analysis, it is found that the active centers are different in the two main views, the studies on the active centers mainly focus on the functional analysis, and the modulation rule on the active centers are rarely reported. Thus, precisely pinpointing the active center in the heterostructure and realizing its accurate modulation are significant for the in‐depth development of MOF‐derived heterostructures as the hosts of S cathode in LSBs. In this regard, we design and synthesize two homologous heterostructures of ZIF‐67‐derived CoSe_2_@CoSe_0.25_S_1.75_ and CoSe_2_@CoS_2_, and confirm that Co at the heterointerface is the primary active center, rather than Co within the individual phases. Meanwhile, it is found that the built‐in electric field is not the deciding factor of Li^+^ storage property of S cathode. Further, we modulate the d‐band center (*ε*_d_) of Co by heterointerface engineering, and demonstrate that *ɛ*_d_ of Co at the heterostructure could be a very significant descriptor for Li^+^ storage of S cathode in LSBs. It is revealed that the adsorption and conversion ability to LiPSs and the discharge‐specific capacity describe a volcano curve as a function of *ɛ*_d_, and the best Li^+^ storage property appears at CoSe_2_@CoSe_0.25_S_1.75_ heterointerface among the five structures considered here (CoSe_2_@CoSe_0.25_S_1.75_, CoSe_2_@CoS_2_, CoSe_0.25_S_1.75_, CoSe_2_, CoS_2_).

## 2 Results and Discussion

### 2.1 Structure Characterization

**Figure 1:**
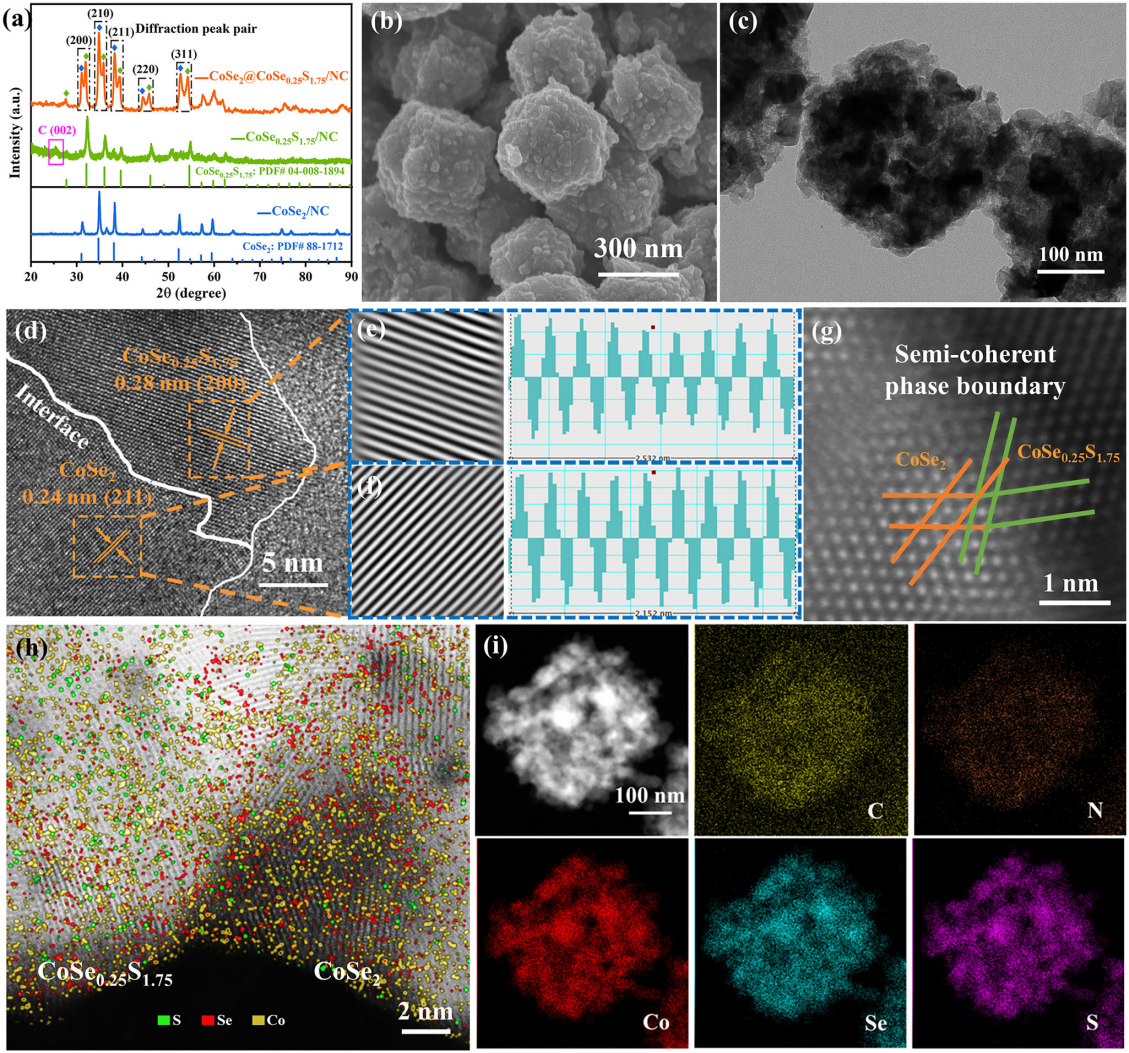
a) XRD patterns of the three hosts. b) SEM image, c) TEM image, d) HRTEM image, e,f) IFFT (Inverse fast Fourier transformation) diagram and corresponding intensity distribution diagram, g) HAADF‐STEM image, h) Near atomic resolution mapping, and i) EDS mapping of CoSe_2_@CoSe_0.25_S_1.75_/NC host.

CoSe_2_@CoSe_0.25_S_1.75_/NC, CoSe_0.25_S_1.75_/NC, and CoSe_2_/NC hosts are synthesized by one‐step solid sintering method, where the ratio of ZIF‐67, S powder, Se powder and the calcination temperature are adjusted. In the XRD (X‐Ray Diffraction) patterns of **Figure** **1**a, these diffraction peaks of CoSe_0.25_S_1.75_/NC and CoSe_2_/NC host are well indexed to cubic CoSe_0.25_S_1.75_ (PDF# 04‐008‐1894) and cubic CoSe_2_ (PDF# 88–1712), respectively, and the diffraction peak corresponding to the (002) plane of NC at 25.3° is also detected. This declares the successful synthesis of the cobalt sulfide or selenide nanoparticles anchored by NC. Notably, in the XRD pattern of CoSe_2_@CoSe_0.25_S_1.75_/NC host, these diffraction peaks assigned to the (200), (210), (211), (220), (311) plane of CoSe_2_ at 31.1°, 34.9°, 38.3°, 44.4°, 52.7°, and these diffraction peaks assigned to the (200), (210), (211), (220), (311) plane of CoSe_0.25_S_1.75_ at 31.9°, 35.9°, 39.3°, 45.8°, 54.2°, are detected simultaneously. This suggests the formation of CoSe_2_ and CoSe_0.25_S_1.75_ complex host, and the characteristic of the twinned diffraction peak pairs with the same plane at a similar diffraction angle suggests their close lattice structure, which can drive the formation of the heterointerface with good lattice compatibility between them. From Scanning Electron Microscope (SEM) images in Figure 1b and Figure S1 (Supporting Information), and Transmission Electron Microscope (TEM) image in Figure 1c, it is seen that the three hosts show similar hollow dodecahedral morphologies with rough surfaces, hierarchical pores and sizes of ≈300 nm, and abundant nanoparticles are uniformly anchored in the NC matrix. Meanwhile, the three hosts show the type‐I and IV isotherm hysteresis loops and the pore size distribution of the mesopores and macropores from 4 to 100 nm, where CoSe_2_@CoSe_0.25_S_1.75_/NC host possesses the maximum pore volume (Figure S2, Supporting Information) with a specific surface area of 26.4 m^2^ g^−1^. These contribute to the uniform loading of S cathode materials, the penetration of these polyhedrons by electrolytes, the host stabilization during charge/discharge process, and the adsorption of host to LiPSs.

HRTEM (High‐resolution TEM) result of CoSe_2_@CoSe_0.25_S_1.75_ nanoparticle (Figure 1d–f) shows that the (211) plane indexed to CoSe_2_ (0.24 nm) and the (200) plane indexed to CoSe_0.25_S_1.75_ (0.28 nm) are observed simultaneously, and the two planes present a feature of atomic lattice contact. Further, from HAADF‐STEM (spherical aberration corrected scanning TEM with high‐angle annular dark‐field) image in Figure 1g, it is observed that the heterointerface manifests a behavior of semi‐coherent phase boundary.^[^ ^20^ ^]^ In the near atomic resolution mapping (Figure 1h), Co element is evenly dispersed on both sides, most of S element is dispersed on the left, and Se element is mainly dispersed on the right of heterointerface. The three phenomena visually display the chemical heterointerface with good compatibility between CoSe_2_ and CoSe_0.25_S_1.75_. In the Energy Dispersive Spectrometer mapping of CoSe_2_@CoSe_0.25_S_1.75_/NC host from TEM (Figure 1i), C and N are evenly dispersed in the whole polyhedron, and Co, Se and S elements are mainly concentrated in nanoparticles, confirming the complex structure of CoSe_2_@CoSe_0.25_S_1.75_ nanoparticles anchored on the NC matrix. Notably, the distribution of Se element is more diffuse than that of S element, which tends to indicate the heterogeneous composition of CoSe_2_ and CoSe_0.25_S_1.75_ rather than CoSe_2_ and CoS_2_.

**Figure 2:**
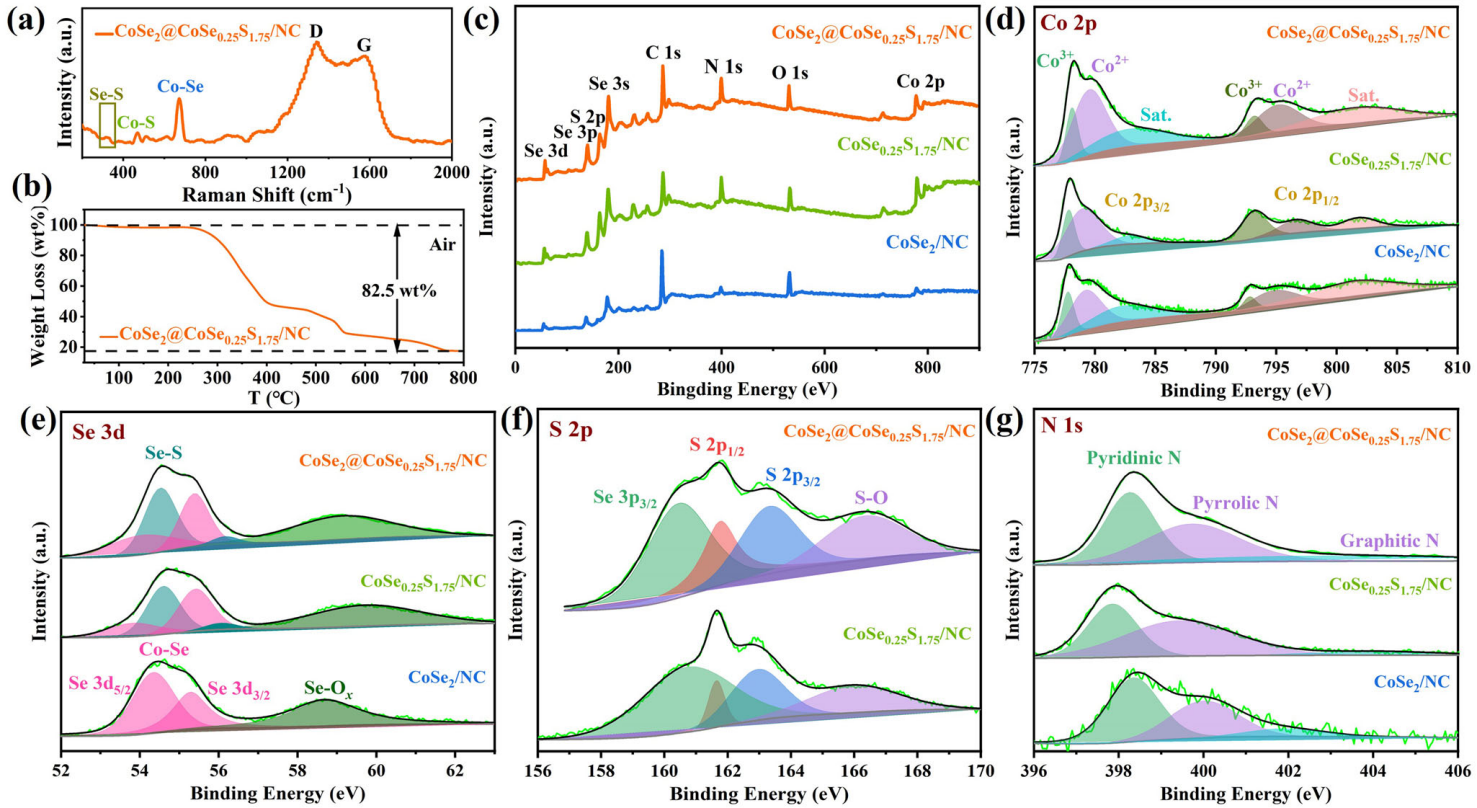
a) Raman spectrum and b) TGA curve of CoSe_2_@CoSe_0.25_S_1.75_/NC host in air. c) Survey, high‐resolution d) Co 2p, e) Se 3d, and g) N 1s XPS spectra of the three hosts. f) High‐resolution S 2p XPS spectra of CoSe_2_@CoSe_0.25_S_1.75_/NC and CoSe_0.25_S_1.75_/NC host.

In the Raman spectrum of CoSe_2_@CoSe_0.25_S_1.75_/NC host (**Figure** **2**a), the Co─Se, Co─S and Se─S bond signals assigned to CoSe_2_@CoSe_0.25_S_1.75_ nanoparticles at 673, 470, and 330 cm^−1^,^[^ ^21^ ^]^ and the D (defective C) and G peak (graphitic C) signals assigned to NC matrix at 1340 and 1570 cm^−1^, are simultaneously collected. This is consistent with the complex structure of CoSe_2_@CoSe_0.25_S_1.75_ and NC. According to the TGA (Thermogravimetric) curve in Figure 2b, the content of CoSe_2_@CoSe_0.25_S_1.75_ is estimated to be 37.5 wt.% in CoSe_2_@CoSe_0.25_S_1.75_/NC host. In the survey XPS (X‐Ray Photoelectron Spectroscopy) spectra (Figure 2c), five elements of Co, Se, S, C, N are simultaneously detected in CoSe_2_@CoSe_0.25_S_1.75_/NC and CoSe_0.25_S_1.75_/NC host, and only four elements of Co, Se, C, N are detected in CoSe_2_/NC host, well matching with their own composite composition. In the high‐resolution Co 2p XPS spectra (Figure 2d), the three hosts show similar peak splitting results, where Co^2+^ and Co^3+^ signals and satellite peaks are revealed simultaneously.^[^ ^22^ ^]^ This is the comprehensive reflection of Co─Se bond, Co─N bond, and surface oxidation. In the high‐resolution Se 3d XPS spectra (Figure 2e), only Co─Se signal is revealed in CoSe_2_/NC host,^[^ ^23^ ^]^ whereas Se─S signal is also observed in CoSe_2_@CoSe_0.25_S_1.75_/NC and CoSe_0.25_S_1.75_/NC host.^[^ ^21c^ ^]^ The Se─S bond mainly comes from CoSe_0.25_S_1.75_. In the high‐resolution S 2p XPS spectra (Figure 2f) of CoSe_0.25_S_1.75_/NC and CoSe_0.25_S_1.75_/NC host, the S 2p_1/2_ and S 2p_3/2_ signals are assigned to S─Co.^[^ ^14^ ^]^ In the high‐resolution N 1s XPS spectra (Figure 2g), the three hosts show similar peak splitting results, where these signals of Pyridinic N, Pyrrolic N, and Graphitic N are revealed simultaneously.^[^ ^24^ ^]^

**Figure 3:**
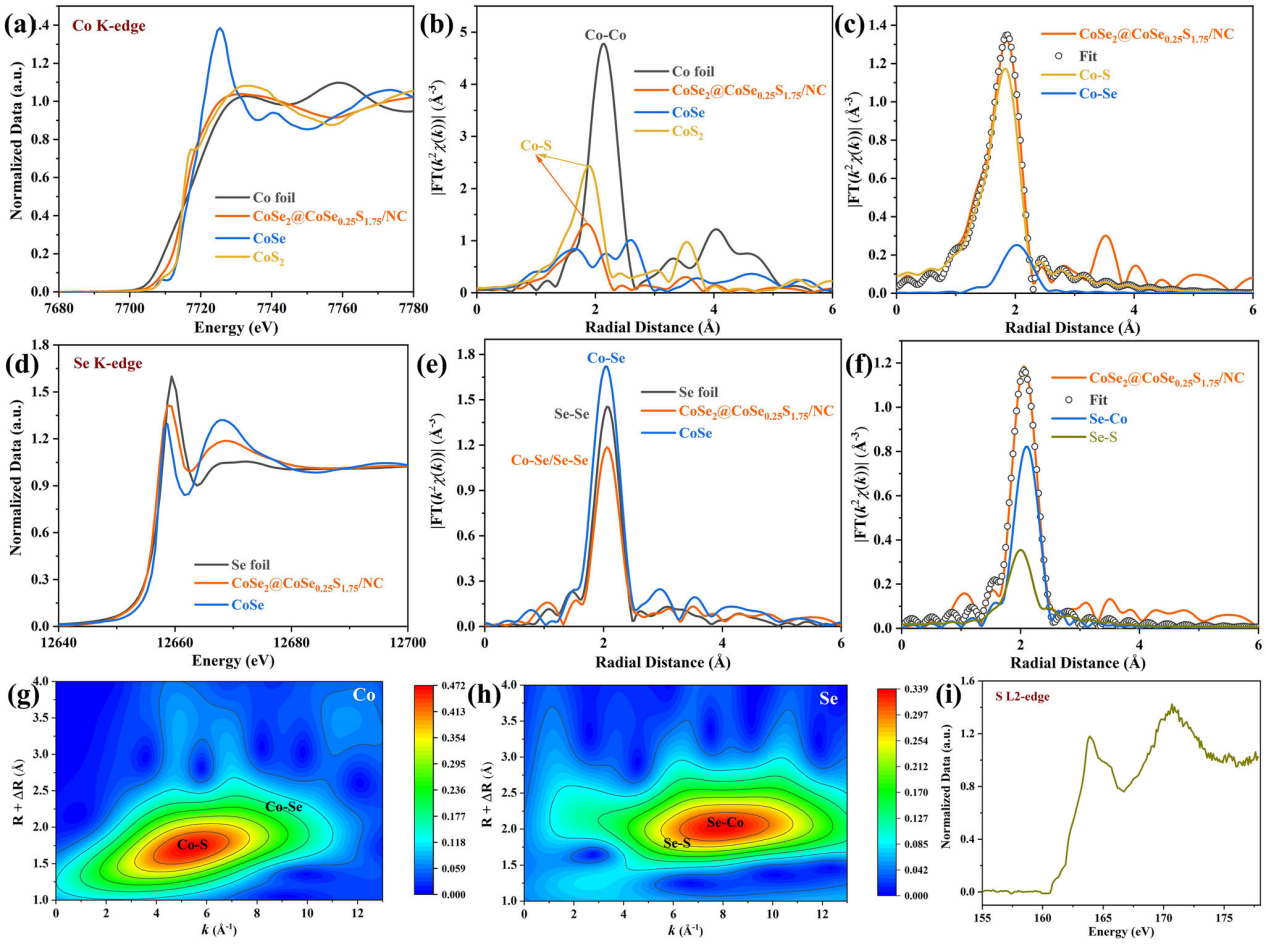
a) Extended XANES, b) FT‐EXAFS, c) Fitted XANES spectrum and g) WT‐EXAFS nephogram of Co, d) Extended XANES, e) FT‐EXAFS, f) Fitted XANES spectrum and h) WT‐EXAFS nephogram of Se, and i) L2‐edge XANES spectrum of S in CoSe_2_@CoSe_0.25_S_1.75_/NC host and reference materials.

From the extended XANES (X‐ray adsorption near‐edge structure) spectra of Co (**Figure** **3**a), it is found that Co K‐edge site lies between Co foil and CoS_2_, and is closer to CoS_2_, indicating the average Co valence slightly lower than +2. This may correspond to the complex coordination of CoSe_2_, CoSe_0.25_S_1.75_, and Co─N bond in CoSe_2_@CoSe_0.25_S_1.75_/NC host. In the FT‐EXAFS (Fourier transform‐extended X‐ray absorption fine structure) spectra (Figure 3b; Figure S3a, Supporting Information), Co─S bond is clearly observed, whose intensity is lower than CoS_2_, indicating its lower coordination number. From the fitted Co K‐edge XANES spectrum (Figure 3c; Figure S4a), the signals of Co─S and Co─Se bonds are simultaneously found. From Se K‐edge XANES spectrum (Figure 3d), it is found that Se K‐edge site lies between Se foil and CoSe, and is closer to Se foil, indicating the Se valence slightly higher than 0. In the FT‐EXAFS spectra (Figure 3e; Figure S3b, Supporting Information), the Se signal is possibly Co─Se or Se─Se bond and inclined to Se─Se bond, whose intensity is lower than CoSe and Se foil, indicating its lower coordination number. This coupled with the low coordination number of Co is highly consistent with the Se‐doped CoS_2_ coordination in CoSe_0.25_S_1.75_. From the fitted Se K‐edge XANES spectrum (Figure 3f; Figure S4b, Supporting Information), the signals of Se─Co and Se─S bonds are simultaneously found. Further quantitative data shown in Table S1 (Supporting Information) presents the similar coordination number and bond length of Co─Se and Se─Co, which coupled with the Se─Se bond indicates the existence of Co─Se alloy. This is possibly relevant to the metallic CoSe_2_, which contributes to enduring the host with good electrical conductivity. Meanwhile, Co─Se exhibits longer bond length and lesser coordination number than Co─S, and Se─S exhibits lesser coordination number than Se─Co, Co─Se, and Co─S. These again demonstrates the existence of CoSe_0.25_S_1.75_. In the wavelet transform (WT)‐EXAFS nephograms of Co (Figure 1g) and Se (Figure 1h), Co─S, Co─Se, Se─Co, and Se─S signals are also detected. Besides, from S L2‐edge XANES spectrum (Figure 1i), relatively simple peaks are observed without peak splitting, indicating the pure Co─S bond.

### 2.2 Electrochemical Performance

**Figure 4:**
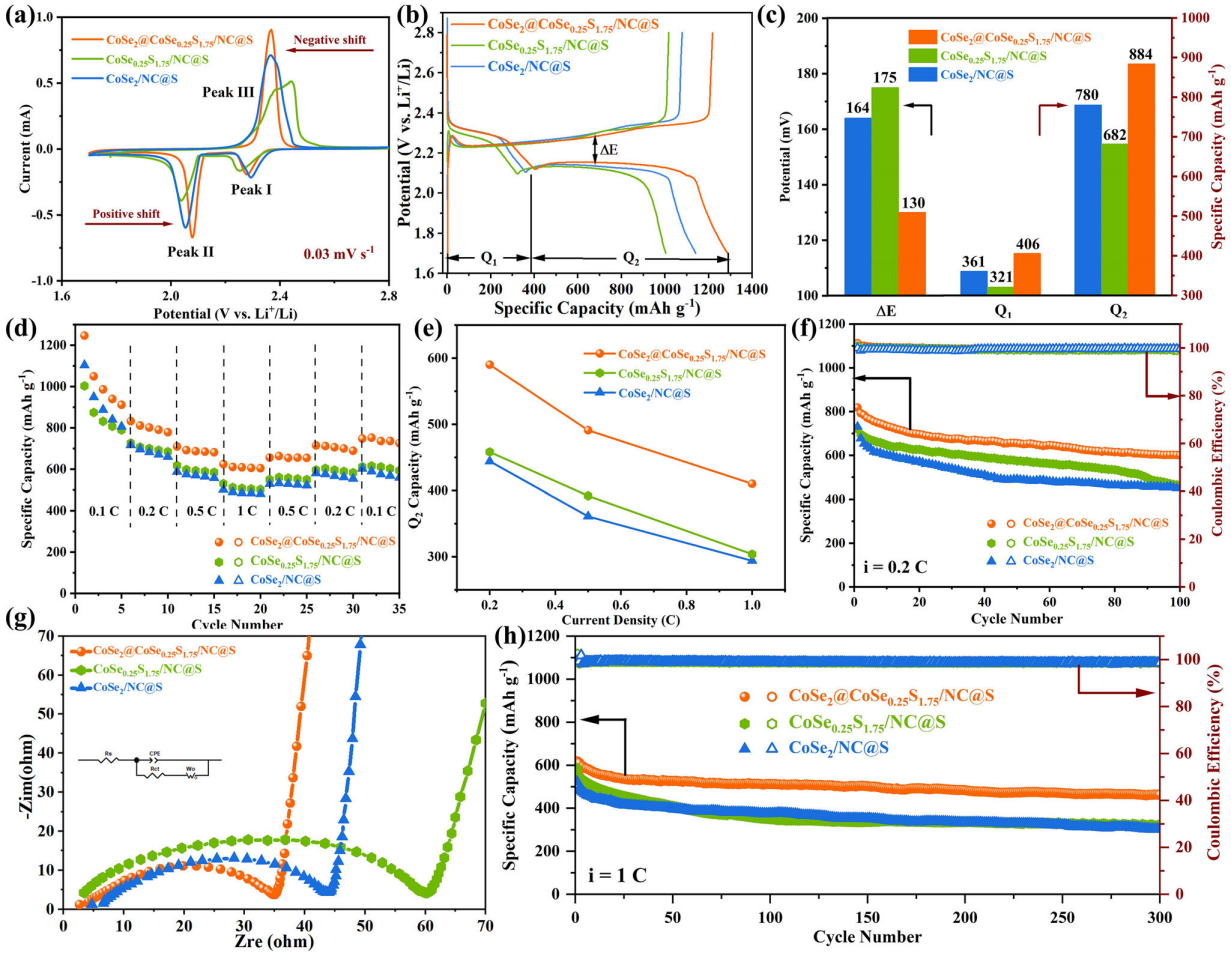
a) Typical CV curves at 0.03 mV s^−1^, b) First charge and discharge profiles at 0.1 C and corresponding c) ∆E values and Capacities, d) Rate capability and corresponding e) Q_2_ capacities at higher rates, f) Cycling performance at 0.2 C, g) EIS plots in the frequency region of 0.1 Hz–10 kHz, and h) Long‐cycling performance at 1 C for the three cathodes. The specific capacity is calculated by using the mass of S active electrode material.

Before testing the electrochemical performance, S powders were injected into the host materials to obtain CoSe_2_@CoSe_0.25_S_1.75_/NC@S, CoSe_0.25_S_1.75_/NC@S, and CoSe_2_/NC@S cathode by melting diffusion method. According to the TGA curve in N_2_ (Figure S5, Supporting Information), the S load is calculated to be ≈70.4 wt.% in CoSe_2_@CoSe_0.25_S_1.75_/NC@S cathode. As shown in **Figure** **4**a, the three cathodes present the representative redox Cyclic Voltammogram (CV) curves, where the two reduction peaks of Peak I and Peak II, and the oxidation peak of Peak III, are indexed to the transformation process of S_8_ (solid) → LiPSs (liquid, long‐chain), Li_2_S_n_ (liquid) → Li_2_S_2_/Li_2_S (solid, short‐chain), and Li_2_S_2_/Li_2_S → S_8_, respectively.^[^ ^25^ ^]^ Notably, CoSe_2_@CoSe_0.25_S_1.75_/NC@S cathode displays the sharpest peak shape and the smallest potential difference between Peak II and Peak III, followed by CoSe_2_/NC@S and CoSe_0.25_S_1.75_/NC@S cathode, indicating its strongest electrochemical reaction ability and the weakest electrochemical polarization. This may be determined by the high electrical conductivity of CoSe_2_. As shown in Figure 4b, the three cathodes present the similar discharge profiles with two distinct platforms and the charge profiles with one platform, which is well corresponding to the CV curves. The potential gap (∆E) between charge and discharge platform, and the first discharge capacity at 0.1 C are calculated to be 130 mV and 1290 mAh g^−1^, 175 mV and 1003 mAh g^−1^, and 164 mV and 1141 mAh g^−1^ for CoSe_2_@CoSe_0.25_S_1.75_/NC@S, CoSe_0.25_S_1.75_/NC@S and CoSe_2_/NC@S cathode (Figure 4C), respectively. This declares the weakest electrochemical polarization and the largest first discharge capacity of CoSe_2_@CoSe_0.25_S_1.75_/NC@S cathode. Especially, it is found that the increment of Q_2_ capacity at low potentials platform is larger than that of Q_1_ capacity at high potentials platform, indicating that the heterostructure significantly improves LiPSs conversion (liquid → solid conversion).

As shown in Figure 4d, CoSe_2_@CoSe_0.25_S_1.75_/NC@S cathode exhibits the best rate performance, and CoSe_0.25_S_1.75_/NC@S cathode exhibits higher capacity at higher rates (0.2C, 0.5C, and 1C) than CoSe_2_/NC@S cathode. Especially, at higher rates, the Q_2_ capacities of CoSe_2_@CoSe_0.25_S_1.75_/NC@S cathode are still prominently higher than other two cathodes (Figure 4e). As shown in Figure S6 (Supporting Information), with the increase in rate, the charge and discharge profiles of CoSe_2_@CoSe_0.25_S_1.75_/NC@S cathode are well retained, especially for the voltage plateaus. Besides, CoSe_2_@CoSe_0.25_S_1.75_/NC@S cathode exhibits the best cycling performance at 0.2 C, where the capacity is up to 600 mAh g^−1^ with a large retention rate of 73.3% (CoSe_0.25_S_1.75_/NC@S: 465 mAh g^−1^, 64.6%: CoSe_2_/NC@S: 450 mAh g^−1^, 61.6%, Figure 4f). Fitting result of Nyquist plots in Figure 4g shows that CoSe_2_@CoSe_0.25_S_1.75_/NC@S cathode also exhibits the smallest charge transfer resistance (R_ct_, 40 Ω, CoSe_0.25_S_1.75_/NC@S: 59 Ω, CoSe_2_/NC@S: 44 Ω), indicating its fast Li^+^ transfer kinetics. Further, CoSe_2_@CoSe_0.25_S_1.75_/NC@S cathode also exhibits the significantly enhanced long‐cycling performance at 1C, where the capacity decay rate is only 0.083% per cycle during 300 cycles (CoSe_0.25_S_1.75_/NC@S: 0.150%; CoSe_2_/NC@S: 0.139%) and the specific capacity is up to 462.5 mAh g^−1^ (CoSe_0.25_S_1.75_/NC@S: 321.5 mAh g^−1^; CoSe_2_/NC@S: 301.7 mAh g^−1^, Figure 4h). Besides, the three cathodes present the stable charge and discharge processes with the coulombic efficiencies of ≈100% during the cycling processes at 0.2 and 1 C.

**Figure 5:**
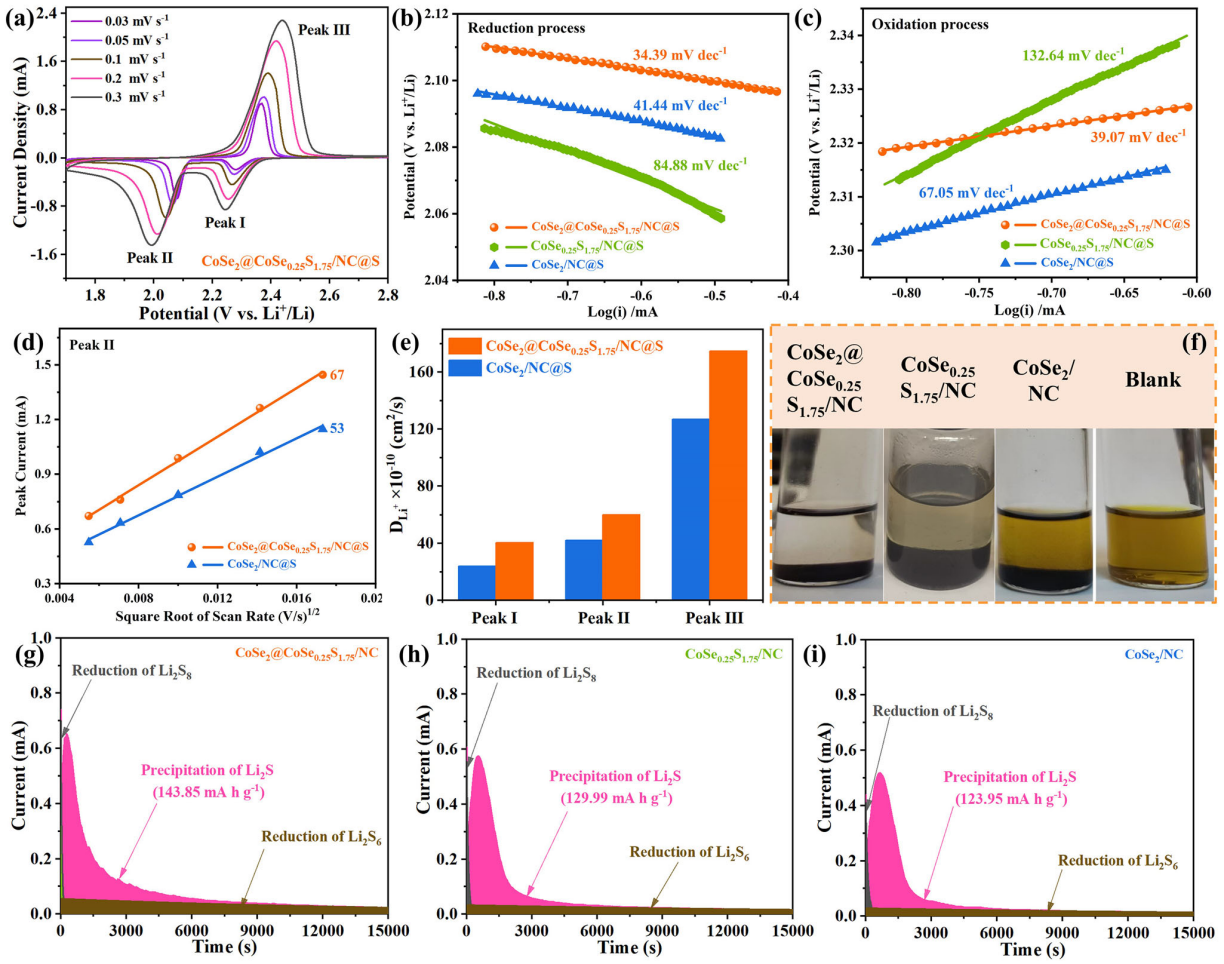
a) CV curves of CoSe_2_@CoSe_0.25_S_1.75_/NC@S cathode at various scan rates. Tafel plots of b) reduction and c) oxidation process for the three cathodes. d) Linear fitting between currents of Peak II and square root of scan rates, and e) D_Li_^+^s of Peak I, II and III for CoSe_2_@CoSe_0.25_S_1.75_/NC@S and CoSe_2_/NC@S cathode. f) Visualized adsorption images of the three hosts to 2.0 mm Li_2_S_6_ solution. Potentiostatic discharge curves of Li_2_S_8_ solution on the surfaces of g) CoSe_2_@CoSe_0.25_S_1.75_/NC, h) CoSe_0.25_S_1.75_/NC, and i) CoSe_2_/NC host.

CV curves are well extended with the increase of the scan rate from 0.03 to 0.3 mV s^−1^ for CoSe_2_@CoSe_0.25_S_1.75_/NC@S (**Figure** **5**a) and CoSe_2_/NC@S (Figure S7a, Supporting Information) cathode, whereas those of CoSe_0.25_S_1.75_/NC@S cathode show obvious polarization (Figure S7b, Supporting Information). This suggests their good current response‐ability to the scan rate of potential and rate capacity. Notably, the Peak II (Figure 5b) and Peak III (Figure 5c) derived Tafel slops of CoSe_2_@CoSe_0.25_S_1.75_/NC@S cathode (34.39 and 39.07 mV dec^−1^) are smallest, followed by CoSe_2_/NC@S (41.44 and 67.05 mV dec^−1^) and CoSe_0.25_S_1.75_/NC@S (84.88 and 132.64 mV dec^−1^) cathode. This declares that CoSe_2_@CoSe_0.25_S_1.75_/NC host possesses the best bidirectional catalytic ability to LiPSs ↔ Li_2_S_2_/Li_2_S conversion process. Meanwhile, the peak current and square root of scan rate present a good linear relationship, and CoSe_2_@CoSe_0.25_S_1.75_/NC@S cathode shows bigger slopes than CoSe_2_/NC@S cathode for Peak I (Figure S7c, Supporting Information), II (Figure 5d), and III (Figure S7d, Supporting Information). Further, it is calculated according to Randles–Sevcik equation (Equation S1, Supporting Information) that CoSe_2_@CoSe_0.25_S_1.75_/NC@S cathode also exhibits higher Li^+^ diffusion coefficients (D_Li_^+^) for the three peaks (Figure 5e). Thus, the heterostructure expedites Li^+^ diffusion kinetics and enhances S redox kinetics. From the visual adsorption experiment (Figure 5f), it is found that CoSe_2_@CoSe_0.25_S_1.75_/NC host displays the best adsorption effect to Li_2_S_6_ (clear and transparent solution), followed by CoSe_0.25_S_1.75_/NC (yellowish solution) and CoSe_2_/NC (dark yellow solution) host. From the nucleation‐growth experiment of Li_2_S at constant potential of 2.02 V, the Li_2_S precipitation capacity is calculated to be 143.85, 129.99 and 123.95 mAh g^−1^ for CoSe_2_@CoSe_0.25_S_1.75_/NC (Figure 5g), CoSe_0.25_S_1.75_/NC (Figure 5h) and CoSe_2_/NC (Figure 5i) host, respectively. As a result, the heterostructure significantly enhances the anchoring and catalytic conversion abilities to LiPSs, and those of CoSe_0.25_S_1.75_/NC host are stronger than CoSe_2_/NC host.

### 2.3 Enhancement Mechanism

**Figure 6:**
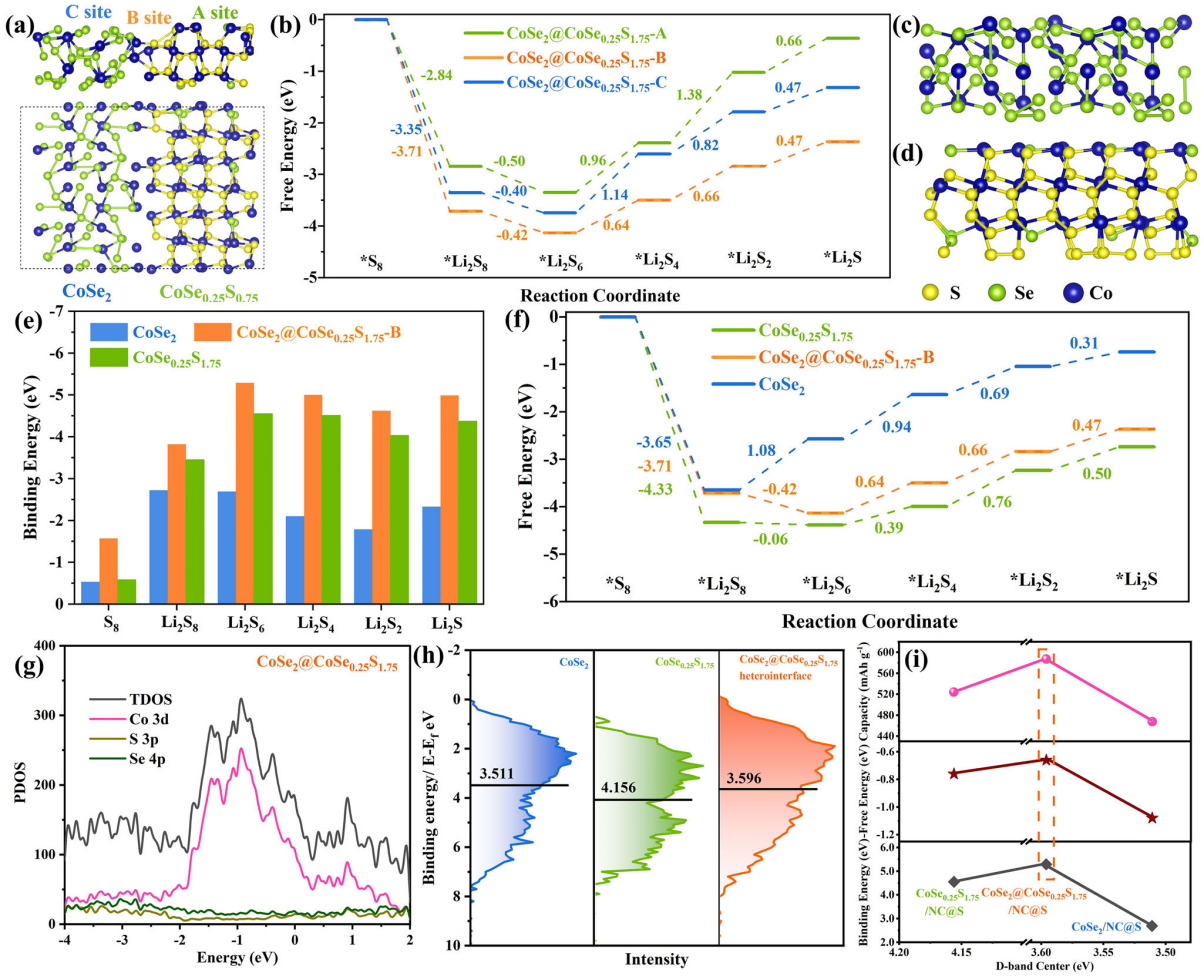
a) Structural model (Upper: side view, Lower: top view) and g) PDOS (projected density of states) of CoSe_2_@CoSe_0.25_S_0.75_ heterostructure. ΔG diagram for the conversion from S_8_ to Li_2_S on b) the three sites (CoSe_0.25_S_0.75_: A site, heterointerface: B site, CoSe_2_: C site) from CoSe_2_@CoSe_0.25_S_0.75_ heterostructure, and f) CoSe_2_ and CoSe_0.25_S_0.75_. Structural model of c) CoSe_2_ and d) CoSe_0.25_S_0.75_ (side view). e) Binding energies for Li_2_S*_x_* and h) VBS (the black bar shows d‐band center), and i) Relationship among binding energy, ‐free energy, the 5^th^ discharge specific capacity at 1 C and *ɛ*_d_ for the three cathodes.

Based on the structural model of CoSe_2_@CoSe_0.25_S_0.75_ heterostructure in **Figure** **6**a, the Gibbs free energy change (ΔG) diagram are obtained in Figure 6b. It is found that the rate‐determining step is the conversion of ^*^Li_2_S_4_ (liquid) to ^*^Li_2_S_2_ (solid) with an uphill ΔG of 1.38 and 0.66 eV on site A (CoSe_0.25_S_0.75_, Figure S8, Supporting Information) and B (heterointerface, Figure S9, Supporting Information), and the conversion of ^*^Li_2_S_6_ to ^*^Li_2_S_4_ on site C with an uphill ΔG of 1.14 eV on site C (CoSe_2_, Figure S10, Supporting Information), respectively. As a result, B site shows the lowest free energy for Li_2_S*_x_* and uphill ΔG (0.66 V) among the three possible sites, revealing that CoSe_2_@CoSe_0.25_S_0.75_ heterointerface is the main active site for LiPSs conversion. Based on the structural model of CoSe_2_ (Figure 6c) and CoSe_0.25_S_0.75_ (Figure 6d), the binding energies and free energies to Li_2_S*_x_* are calculated. As shown in Figure 6e, CoSe_2_@CoSe_0.25_S_0.75_ heterostructure exhibits the largest binding energies for Li_2_S*_x_*, indicating its strongest capture capacity for Li_2_S*_x_*, followed by CoSe_0.25_S_0.75_ and CoSe_2_. As found in Figure 6f, CoSe_0.25_S_0.75_ presents the same rate‐determining step (uphill ΔG: 0.76 eV) with CoSe_2_@CoSe_0.25_S_0.75_ heterointerface (Figure S11, Supporting Information), whereas that of CoSe_2_ is the conversion of ^*^Li_2_S_8_ (liquid) to ^*^Li_2_S_6_ (liquid) with an uphill ΔG of 1.08 eV (Figure S12, Supporting Information). This may be highly related to the high electrical conductivity of CoSe_2_.

Thus, CoSe_2_@CoSe_0.25_S_0.75_ heterostructure possesses the strongest adsorption and catalytic conversion ability to LiPSs, followed by CoSe_0.25_S_0.75_ and CoSe_2_. Notably, CoSe_2_ shows a unique catalytic conversion path with different rate‐determining step. Further, it is calculated that the d‐band of Co contributes most of TDOS rather than the p‐bands of S and Se (Figure 6g; Figure S13, Supporting Information), which reveals that Co is the specific active center. The d‐band center (*ε*_d_) of Co is then calculated to be 3.596, 3.511, and 4.156 eV by Valence band spectra (VBS) for CoSe_2_@CoSe_0.25_S_0.75_ heterointerface, CoSe_2_ and CoSe_0.25_S_0.75_, respectively (Figure 6h). As presented in Figure 6i, the upward shift of *ε*_d_ promotes the hybridization between Co active sites and LiPSs, thus enhancing the adsorption and catalytic conversion ability to LiPSs. However, the excessive upward shift of *ε*_d_ would cause the decrease of the adsorption and catalytic conversion ability to LiPSs. Thus, the CoSe_2_@CoSe_0.25_S_1.75_/NC@S cathode with moderate *ε*_d_ exhibits the largest discharge‐specific capacity.

### 2.4 Modulation Rule

**Figure 7:**
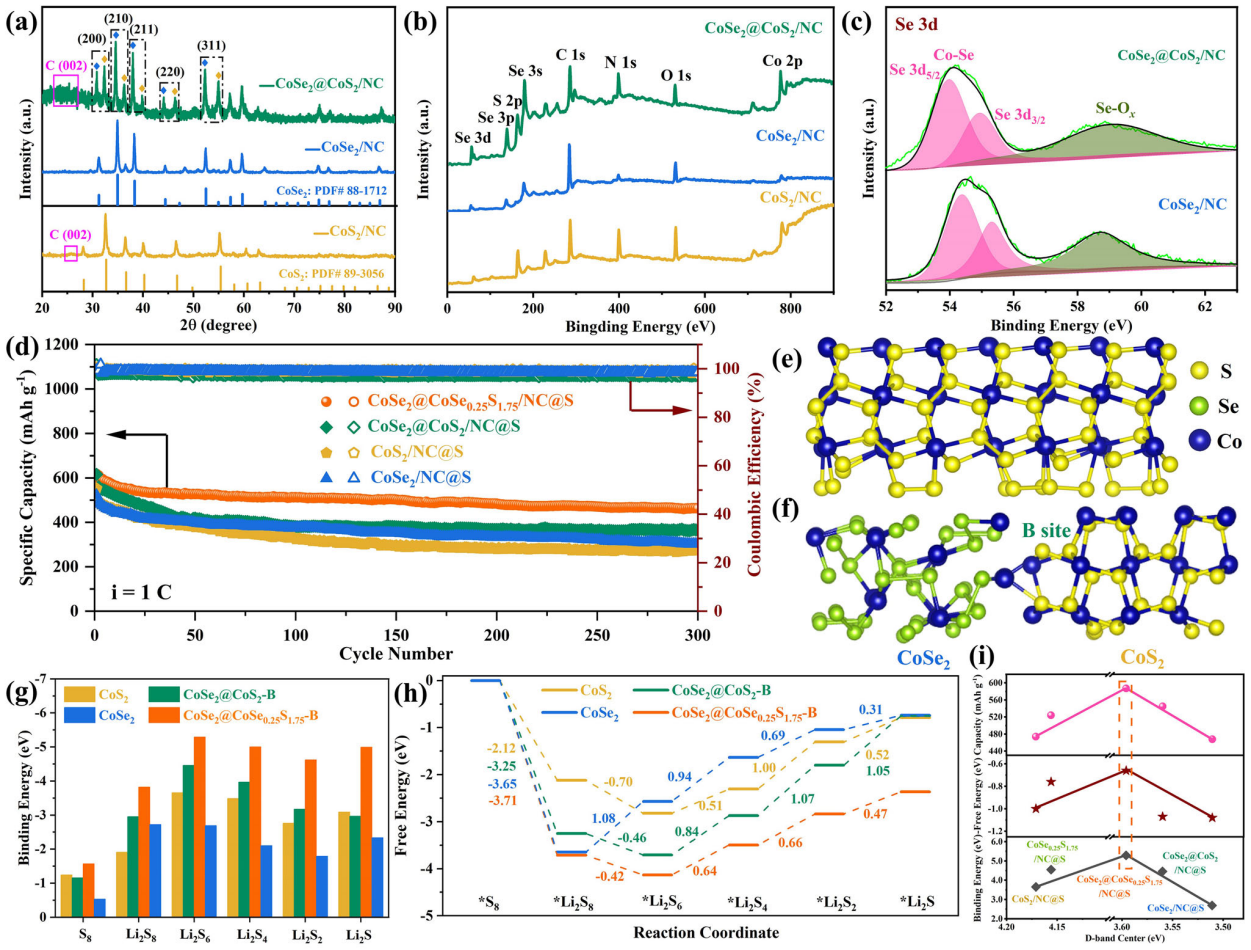
a) XRD patterns and b) Survey XPS spectra of CoSe_2_@CoS_2_/NC, CoSe_2_/NC, and CoS_2_/NC host. c) High‐resolution Se 3d XPS spectra of CoSe_2_@CoS_2_/NC and CoSe_2_/NC host. d) Long‐cycling performance at 1 C, g) Adsorption energies for Li_2_S*_x_*, and h) ΔG diagram for the conversion from S_8_ to Li_2_S on CoSe_2_@CoSe_0.25_S_0.75_‐B, CoSe_2_@CoS_2_‐B, CoSe_2_ and CoS_2_ site. Structural model of e) CoS_2_ and f) CoSe_2_@CoS_2_ (side view). i) Relationship among binding energy, ‐free energy, the 5th discharge specific capacity at 1 C and *ɛ*_d_ for the five cathodes.

To better confirm the advantage of CoSe_2_@CoSe_0.25_S_0.75_ heterointerface and further reveal the modulation rule of the *ɛ*_d_ of Co on LiPSs conversion, CoSe_2_@CoS_2_/NC and CoS_2_/NC hosts were synthesized. As shown in **Figure** **7**a, these diffraction peaks of CoS_2_/NC host are well indexed to cubic CoS_2_ (PDF# 89–3056), and the diffraction peak of NC at 25.7° and 25.1° is also detected in the XRD patterns of CoS_2_/NC and CoSe_2_@CoS_2_/NC host, respectively. Especially, CoSe_2_@CoS_2_/NC host also presents the diffraction pattern characteristic of CoS_2_ and CoSe_2_ twinned diffraction peak pairs, suggesting the formation of the heterointerface with good lattice compatibility between them. As shown in Figure S14 (Supporting Information), CoSe_2_@CoS_2_/NC and CoS_2_/NC hosts also show similar hollow dodecahedral morphologies. Survey (Figure 7b), high‐resolution Co 2p (Figure S15a, Supporting Information), Se 3d (Figure 7c), S 2p (Figure S15b, Supporting Information), and N 1s (Figure S15c, Supporting Information) XPS spectra are well coincident with the corresponding composition of CoSe_2_@CoS_2_/NC and CoS_2_/NC host. As shown in Figure 7d, CoSe_2_@CoS_2_/NC@S cathode presents better cycling performance at 1 C than CoSe_2_/NC@S and CoS_2_/NC@S cathode, whereas that is worse than CoSe_2_@CoSe_0.25_S_1.75_/NC@S cathode. Meanwhile, at the low rate of 0.2 C, the cycling performance of CoSe_2_@CoS_2_/NC@S cathode is still better than CoSe_2_/NC@S and CoS_2_/NC@S cathode, and worse than CoSe_2_@CoSe_0.25_S_1.75_/NC@S cathode (Figure S16, Supporting Information). As shown in Figure S17 (Supporting Information), CoSe_2_@CoS_2_/NC@S cathode presents well electrochemical kinetics to scan rates, whereas CoS_2_/NC@S cathode presents obvious polarization. This is in good agreement with the good electrical conductivity of CoSe_2_.

DFT (Density Functional Theory) calculation results according to the structural models of CoS_2_ (Figure 7e; Figure S18, Supporting Information) and CoSe_2_@CoS_2_ (Figure 7f; Figure S19, Supporting Information) show that CoSe_2_@CoS_2_ heterointerface exhibits larger adsorption energies for Li_2_S*_x_* than CoS_2_ and CoSe_2_ (Figure 7g). Meanwhile, CoSe_2_@CoS_2_ heterointerface and CoS_2_ also present the rate‐determining step of the conversion of ^*^Li_2_S_4_ to ^*^Li_2_S_2_ with an uphill ΔG of 1.07 and 1.00 eV, respectively. As a result, CoSe_2_@CoS_2_ heterointerface contributes to strengthening the adsorption ability, but is against the improvement of the conversion ability for various LiPSs intermediate species with respect to CoS_2_ and CoSe_2_ (Figure 7h). Notably, CoSe_2_@CoSe_0.25_S_0.75_ heterostructure presents stronger adsorption and conversion ability to LiPSs than CoSe_2_@CoS_2_ heterostructure. It is also found that CoS_2_ possesses stronger adsorption and conversion abilities than CoSe_2_, and the doping of Se to CoS_2_ (CoSe_0.25_S_0.75_) significantly strengthens the adsorption and conversion ability of CoS_2_. Further, Co is confirmed to be still the specific active center at CoSe_2_@CoS_2_ heterointerface (Figure S20a, Supporting Information) and CoS_2_ (Figure S20b, Supporting Information), whose *ɛ*_d_ is calculated to be 3.560 and 4.171 eV, respectively. As revealed in Figure 7i, the adsorption ability, conversion ability and discharge specific capacity describe a volcano curve as a function of *ɛ*_d_, and the best cathode is found to be CoSe_2_@CoSe_0.25_S_1.75_/NC@S among the five cathodes of CoSe_2_@CoSe_0.25_S_1.75_/NC@S, CoSe_2_@CoS_2_/NC@S, CoSe_0.25_S_1.75_/NC@S, CoSe_2_/NC@S and CoS_2_/NC@S. Thus, the *ɛ*_d_ of Co in the heterostructure could be a very significant descriptor for Li^+^ storage of S cathode in LSBs.

In addition, the work function (W) of CoSe_2_, CoSe_0.25_S_0.75_, and CoS_2_ is calculated to be 4.82 (Figure S21a, Supporting Information), 5.25 (Figure S21b, Supporting Information), and 5.44 (Figure S21c, Supporting Information) eV, respectively. Higher *E*_f_ and larger W of CoSe_0.25_S_0.75_ and CoS_2_ than CoSe_2_ potentially induce the electron transfer from CoSe_2_ to CoSe_0.25_S_0.75_ and CoS_2_ with a built‐in electric field generation from CoSe_2_ to CoSe_0.25_S_0.75_ (Figure S22, Supporting Information) and CoS_2_ at the CoSe_2_@CoSe_0.25_S_0.75_ and CoSe_2_@CoS_2_ heterointerface, respectively. Further, the W of CoSe_2_@CoSe_0.25_S_0.75_ and CoSe_2_@CoS_2_ heterointerface is calculated to be between that of CoSe_0.25_S_0.75_ and CoSe_2_ (5.10 eV, Figure S23a, Supporting Information), and CoS_2_ and CoSe_2_ (5.23 eV, Figure S23b, Supporting Information), respectively. This coupled with the differential charge density result (Figure S24, Supporting Information) confirms the occurrence of electron transfer between them. The built‐in electric field contributes to driving the electron transfer between host and Li_2_S*_x_*, thus resulting in the enhanced conversion ability of LiPSs. Notably, although CoSe_2_@CoS_2_ heterointerface has bigger built‐in electric field than CoSe_2_@CoSe_0.25_S_0.75_ heterointerface, its adsorption and conversion ability are weaker, which suggests that the built‐in electric field is not the deciding factor for Li^+^ storage property of S cathode. Besides, XAFS, XPS, and DFT results show that there is no formation of interfacial chemical bonds at CoSe_2_@CoSe_0.25_S_0.75_ heterointerface.

## 3 Conclusion

The two homologous hetero‐hosts of ZIF‐67‐derived CoSe_2_@CoSe_0.25_S_1.75_/NC and CoSe_2_@CoS_2_/NC are successfully synthesized. The characteristic of the twinned diffraction peak pairs drives the formation of the heterointerface with a behavior of semi‐coherent phase boundary. Co at the heterointerface is proved to be the primary active center, rather than Co within the individual phases. The *ɛ*_d_ of Co in the heterostructure could be a very significant descriptor for the adsorption ability, conversion ability and discharge specific capacity those describe a volcano curve as a function of *ɛ*_d_, and the best cathode is found to be CoSe_2_@CoSe_0.25_S_1.75_/NC@S. The capacity is up to 600 mAh g^−1^ with a large retention rate of 73.3% at 0.2 C after 100 cycles, and the capacity decay rate is only 0.083% per cycle at 1 C during 300 long‐cycling process. Besides, CoSe_2_ shows a unique catalytic conversion path (Rate‐determining step: ^*^Li_2_S_8_ → ^*^Li_2_S_6_) that is different from the other four materials (Rate‐determining step: ^*^Li_2_S_4_ → ^*^Li_2_S_2_). Doping of Se to CoS_2_ (CoSe_0.25_S_0.75_) can significantly strengthen the adsorption and conversion ability of CoS_2_. The built‐in electric field is not the deciding factor for Li^+^ storage property of S cathode in CoSe_2_@CoSe_0.25_S_1.75_/NC and CoSe_2_@CoS_2_/NC hetero‐hosts.

## Conflict of Interest

The authors declare no conflict of interest.

## Supporting information



Supporting Information

## Data Availability

The data that support the findings of this study are available in the supplementary material of this article.
