# Supplementary material for: Unlocking Modulation Rule of Heterointerface Engineering Induced D‐Band Center on Polysulfides Conversion in Lithium–Sulfur Batteries
Source: Adv Sci (Weinh). 2025 Mar 6;12(17):2501940. doi: 10.1002/advs.202501940 (PMC12061276; doi:10.1002/advs.202501940)
Supplement: Supplementary file 1 — Supporting Information [file ADVS-12-2501940-s001.docx]

Supplementary Materials for

**Unlocking Modulation Rule of** **Heterointerface Engineering Induced D-Band Center on** **Polysulfides Conversion in Lithium-Sulfur Batteries**

*Wenbin Li,^§,1,2^ Ni Wang^§,1,3^, Jingjie Pei^§,1,2^, Dongyan Li ^1,2^, Guiqiang Cao ^1,2^, Ruixian Duan ^1,2^, Jingjing Wang, ^1,2^ Xifei Li ^1,2,4 *^*

*^1^Institute of Advanced Electrochemical Energy and School of Materials Science and Engineering, Xi’an University of Technology, Xi’an, 710048, China*

*^2^Shaanxi Engineering Research Center of Key Materials for Lithium/Sodium-ion Batteries, Shaanxi International Joint Research Center of Surface Technology for Energy Storage Materials, Key Laboratory of Advanced Batteries Materials for Electric Vehicles of China Petroleum and Chemical Industry Federation, Xi’an 710048, China*

*^3^Xi’an North Huian Chemical Industries Company, Xi’an 710302, Shaanxi, China*

*^4^Guangdong Yuanneng Technologies Co Ltd, Foshan, Guangdong, 528223, China*

*^§^These authors contributed equally to this work*

*E-mail address: xfli2011@hotmail.com (X. Li)*

# Experimental section

**Synthesis of ZIF-67 precursor**

The ZIF-67 precursor was prepared by a minor adjustment according to the previous work. ^[1]^ Typically, 0.9 g of cobalt nitrate hexahydrate (Co(NO)_3_⸱6H_2_O, Sinopharm, AR) was dissolved in 6 mL of deionized water (DI) called solution A. Then, 11.0 g of 2-methylimidazole (2-MeIm, Sinopharm, 99%) was dissolved in 20 mL DI water called solution B. After that, A solution was quickly poured into B solution under magnetic stirring, and then the mixture was stood at room temperature for 24 h after continuously stirred for 10 min. Finally, the purple precipitates (ZIF-67) were centrifuged and washed thoroughly with methanol three times, and vacuum dried overnight at 60 ^o^C for further use.

**Synthesis of CoSe_2_@CoSe_0.25_S_1.75_/NC, CoSe_0.25_S_1.75_/NC, CoSe_2_/NC, CoSe_2_@CoS_2_/NC, CoS_2_/NC host**

ZIF-67 precursor, Se powder (Sinopharm, CP) and sublimed sulfur (aladdin, AR) were firstly mixed by hand grind with a mass ratio of 1: 2: 1. After that, the mixed powders were directly calcined at 450 ^o^C for 2 h at a heating rate of 2 ^o^C/min, which were then naturally cooled to 25 ^o^C under flowing of nitrogen gas to obtain CoSe_2_@CoSe_0.25_S_1.75_/NC host. CoSe_0.25_S_1.75_/NC host was synthesized under the mass ratio of 1: 4: 4 (ZIF-67 precursor: Se powder: sublimed sulfur). CoSe_2_/NC host was synthesized by removing the sublimed sulfur. CoS_2_/NC host was synthesized by removing the Se powder and decreasing the calcination temperature to 400 ^o^C. CoSe_2_@CoS_2_/NC host was synthesized by calcining the mixed powders of CoSe_2_/NC and sublimed sulfur with a mass ratio of 1: 2 at 450 ^o^C for 2 h at a heating rate of 2 ^o^C/min.

**Preparation of** **CoSe_2_@CoSe_0.25_S_1.75_/NC@S, CoSe_0.25_S_1.75_/NC@S, CoSe_2_/NC@S, CoSe_2_@CoS_2_/NC@S, CoS_2_/NC@S**

The frequently-used melt diffusion technology was employed to impregnate the S powder to the above hosts, and thus obtaining the CoSe_2_@CoSe_0.25_S_1.75_/NC@S, CoSe_0.25_S_1.75_/NC@S, CoSe_2_/NC@S, CoSe_2_@CoS_2_/NC@S and CoS_2_/NC@S cathode materials. Concretely, the above hosts were hand-milled with S power for 15 ~ 20 min at the mass ratio of 7: 3. After that, the uniform mixtures were heated to 155 ^o^C (heating rate: 5 ^o^C min^-1^) that kept for 12 h in a tubular furnace populated with Ar flow. Finally, the above cathode materials were obtained after naturally cooling to room temperature.

**Structural characterization**

The morphology was seen using FESEM with gold spraying (Field Emission Scanning Electron Microscope, ZEISS, MERLIN Compact) and FETEM (Field Emission Transmission Electron Microscope, JEOL JEM_2_100PLUS) where the powder particles were stabilized with carbon support films. The crystal phase was characterized by XRD (X-Ray Diffraction, Lab X XRD-7000, Cu Kα X-ray source, Scanning speed: 5 ^o^min^-1^, 2θ = 10 ~ 90 ^o^) and FETEM. The elemental composition and valence bond were analyzed by XPS (X-Ray Photoelectron Spectroscopy, Thermo Fisher Escalab 250xi, Radiation: monochromatic Al Kα) and Raman (Thermo Fisher DXRxi 532, Excitation laser wavelength: 532 nm). The specific surface area and porous structure were gained by BET (Brunauer-Emmet-Teller, Micromeritics ASAP_2_460) test with nitrogen adsorption at the pressure range of 0 ~ 1 e^-7^. The TGA (Thermogravimetric analysis, NETZSCH STA 449 F_5_) curve was collected by a thermal analyzer (Heating rate: 10 ^o^C min^-1^). HAADF-STEM (high-angle annular dark-field-scanning TEM, thermalfisher scientific titan themsis Z) equipped with a field emission gun at 300 kV was utilized to refine the atomic arrangement of heterointerface, and HAADF-mapping was utilized to confirm the elemental composition of heterogeneous nanoparticles.

Co and Se K-edge, and S L2-edge analyses were performed with Si(111) crystal monochromators at the BL14W1 beamlines at the Shanghai Synchrotron Radiation Facility (SSRF) (Shanghai, China). Before the analysis at the beamline, samples were pressed into thin sheets with 1 cm in diameter and sealed using Kapton tape film. The XAFS spectra were recorded at room temperature using a 4-channel Silicon Drift Detector (SDD) Bruker 5040. Co and Se K-edge extended X-ray absorption fine structure (EXAFS) spectra were recorded in transmission mode. Negligible changes in the line-shape and peak position of Co and Se *K*-edge XANES spectra were observed between two scans taken for a specific sample. The XAFS spectra of these standard samples (Co foil, Se foil, CoSe and CoS_2_) were recorded in transmission mode. The XAFS data were processed according to the standard procedures using the Athena module implemented in the IFEFFIT software packages. The EXAFS spectra were obtained by subtracting the post-edge background from the overall absorption and then normalizing with respect to the edge-jump step. Subsequently, the *χ(k)* data were Fourier transformed to real (R) space using a hanning windows (*dk* = 1.0 Å^−1^) to separate the EXAFS contributions from different coordination shells. To obtain the quantitative structural parameters around central atoms, least-squares curve parameter fitting was performed using the ARTEMIS module of IFEFFIT software packages. ^[2]^

All the calculations were performed in the framework of the density functional theory (DFT) with the projector augmented plane-wave method, as implemented in the Vienna ab initio simulation package. ^[3]^ The generalized gradient approximation proposed by Perdew-Burke-Ernzerhof (PBE) was selected for the exchange-correlation potential. ^[4]^ The cut-off energy for plane wave was set to 500 eV. The energy criterion was set to 10^−5^ eV in iterative solution of the Kohn-Sham equation. All the structures were relaxed until the residual forces on the atoms had declined to less than 0.02 eV/Å. To avoid interlaminar interactions, a vacuum spacing of 20 Å was applied perpendicular to the slab.

**Electrochemical measurements**

To obtain the CR2032 button battery, the cathode plates, anode plates and separators were laminated and encapsulated with the injection of 40 μL of electrolytes in an Ar-populated glovebox, where the contents of H_2_O and O_2_ were strictly controlled to < 1 ppm. The black slurries were modulated by uniformly mixing 70 wt% of the above cathode materials, 20 wt% of Super P (conducting agent) and 10 wt% of PVDF (Polyvinylidene Fluoride, binder) in the NMP (N-Methyl-2-Pyrrolidone, solvent). The slurries were then coated onto Al foils, which were dried at 60 ^o^C for 12 h in a vacuum drying oven. After that, the cathode plates were obtained by cutting the Al foil containing the black slurry into a circle disk with the diameter of 12 mm. The anode plates were Metal Li and the separators were Celgard 2500 PP (Polypropylene) fiber film. The electrolytes were the mixed solution of 1 M LiTFSI (Bis (Trifluoromethane) Sulfonimide Lithium Salt) in DOL (Dioxolame) and DME (Dimethyl Ether, volume ratio 1:1) containing 1 wt% LiNO_3_.

The galvanostatic cycle and rate performances were measured in a voltage range of 1.7 ~ 2.8 V (*vs*. Li/Li^+^) by a charge/discharge system (CT-4008-5V_1_0mA-164 Neware). The CV (Cyclic Voltammogram) curve (1.7 ~ 2.8 V) and EIS (Electrochemical Impedance Spectroscopy, 0.1 to 100 kHz) were collected by an electrochemical workstation (Princeton Applied Research VersaSTAT 4). The Li^+^ diffusion coefficients were calculated by CV curves at different scan rates according to Randles-Sevcik equation:

I_peak_ = (2.69×10^5^) n^1.5^AD_Li_^0.5^υ^0.5^C_Li_ (1)

I_peak_ stands for the peak current (A); n stands for the electron number in the electrochemical reaction (n = 2 in this work); A stands for the geometrical area of the electrode (A = 1.13 cm^2^ in this work); D_Li_ stands for the Li^+^ diffusion coefficient; υ stands for the scan rate of CV test and C_Li_ stands for the concentration of Li^+^ in the electrolyte (C_Li_ = 1 mol∙L^-1^ or 0.001 mol∙mL^-1^). ^[5]^

In order to perform the nucleation-growth test of Li_2_S, the battery was assembled according to the sequence of Metal Li plate (anode), the electrolyte of 1 M LiTFSI in DOL and DME (volume ratio 1:1) containing 1 wt% LiNO_3_, Celgard 2500 PP fiber film, the electrolyte of 0.3 M Li_2_S_8_, and cathode plate. The cathode plate was composed of 90 wt% of host materials and 10% PVDF, which was dispersed in the NMP and then coated onto an Al foil. The electrolyte of 0.3 M Li_2_S_8_ was dispensed by uniformly dissolving the Li_2_S_8_, sublimed sulfur, and LiTFSI with the molar ratio of 1: 7: 4 in tetraethylene glycol dimethyl ether solution. The battery was tested at a constant potential of 2.02 V by a charge/discharge system (CT-4008-5V_1_0mA-164 Neware) to obtain the current-discharge time curves, where the battery was firstly discharged to 2.06 V at a constant current of 0.112 mA and then discharged to 10^-2^ mA at a constant potential of 2.02 V. The visual adsorption test was carried out by seeing the color change of 2.0 mM Li_2_S_6_ solution after addition 50 mg host materials into 3 mL solution for 6 h. Concretely, the Li_2_S and S with the molar ratio of 1: 5 were dissolved in DOL/DME mixed solution. After magnetic stirring for 24 h at 60 ^o^C, 0.2 M Li_2_S_6_ solution was obtained. Before adsorption test, the above Li_2_S_6_ solution was diluted to 2.0 mM.


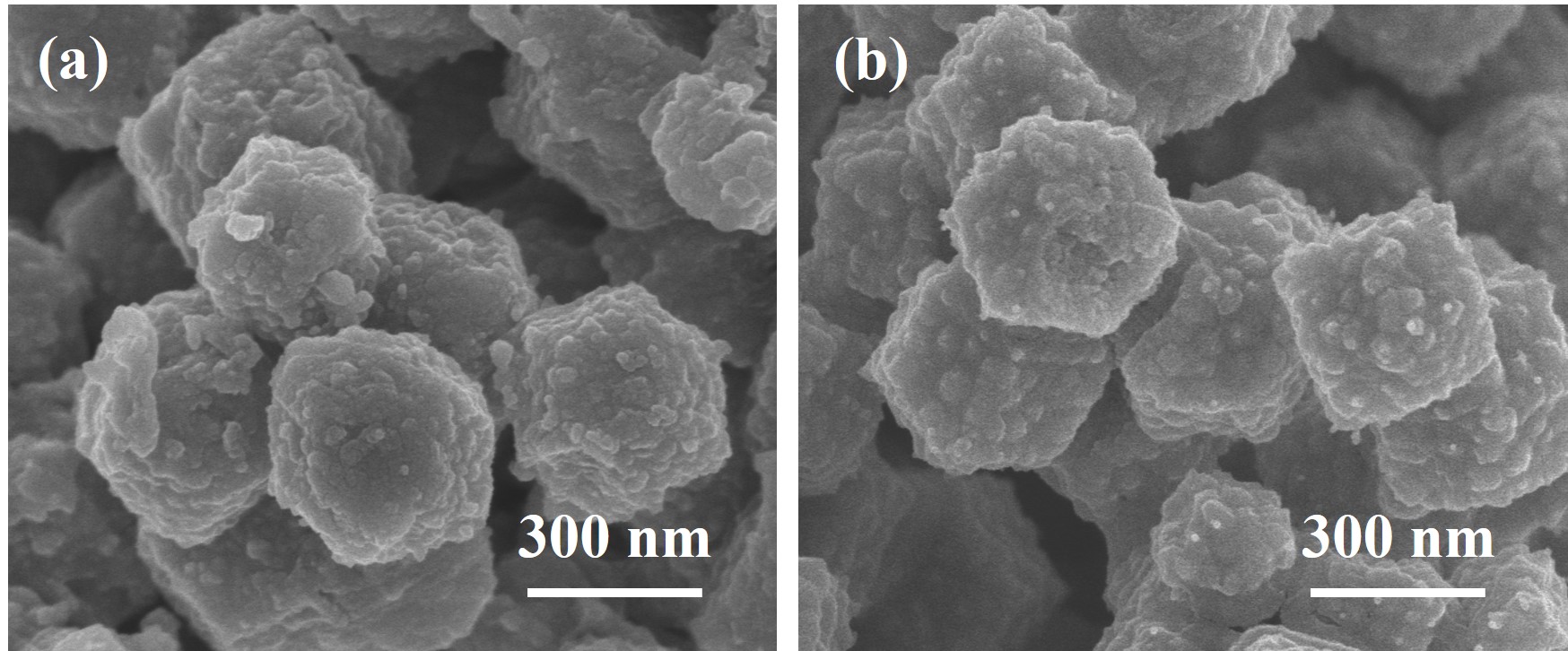


**Fig. S1** SEM image of (a) CoSe_0.25_S_1.75_/NC and (b) CoSe_2_/NC host.


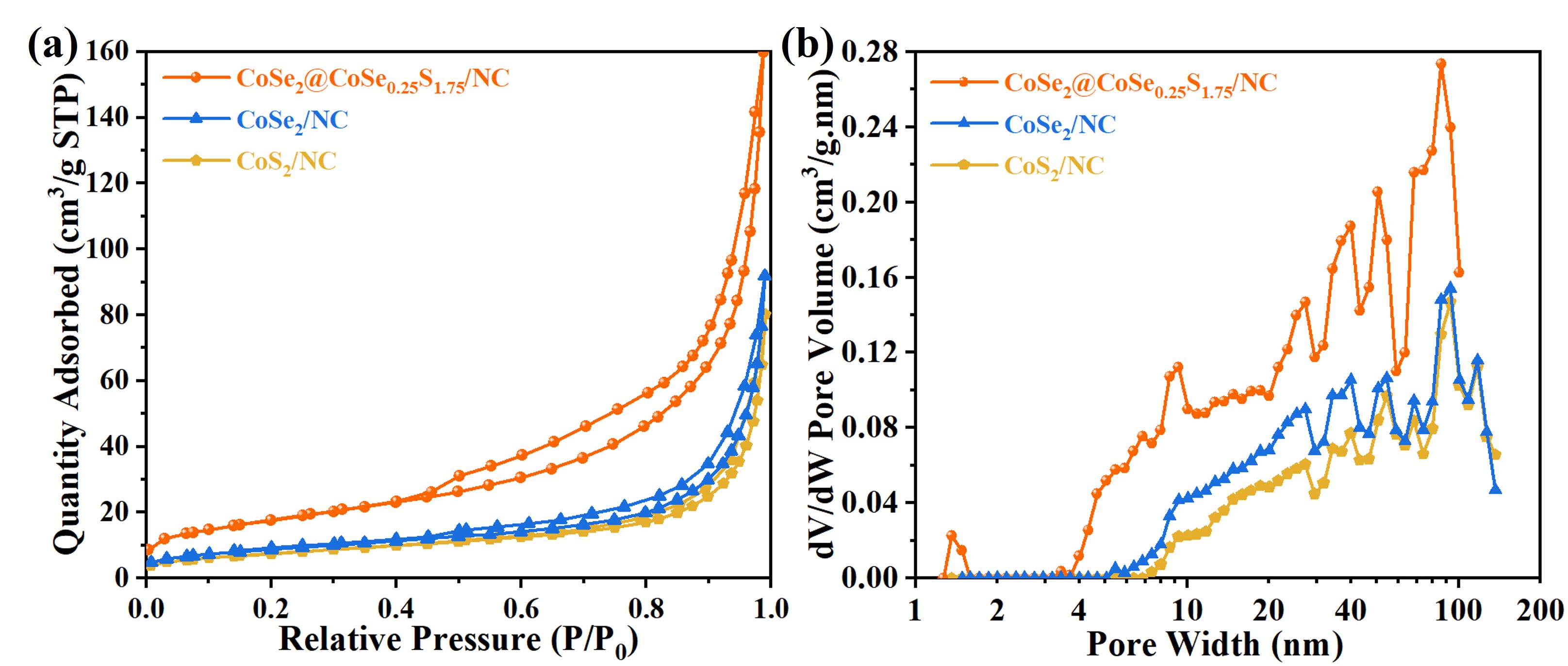


**Fig. S2** (a) Nitrogen adsorption/desorption isotherms and (b) Pore size distributions of CoSe_2_@CoSe_0.25_S_1.75_/NC, CoSe_2_/NC and CoS_2_/NC host.


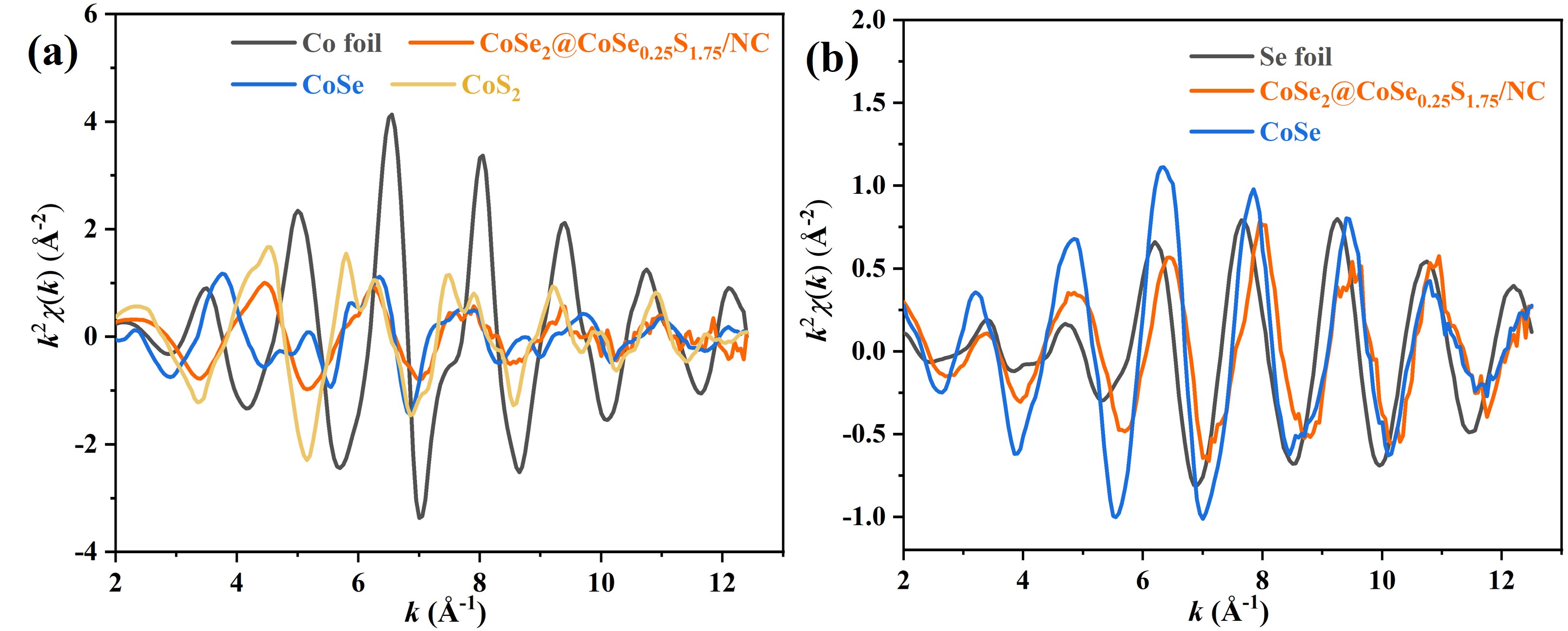


**Fig. S3** K3 space spectra of (a) Co and (b) Se in CoSe_2_@CoSe_0.25_S_1.75_/NC host and reference materials.


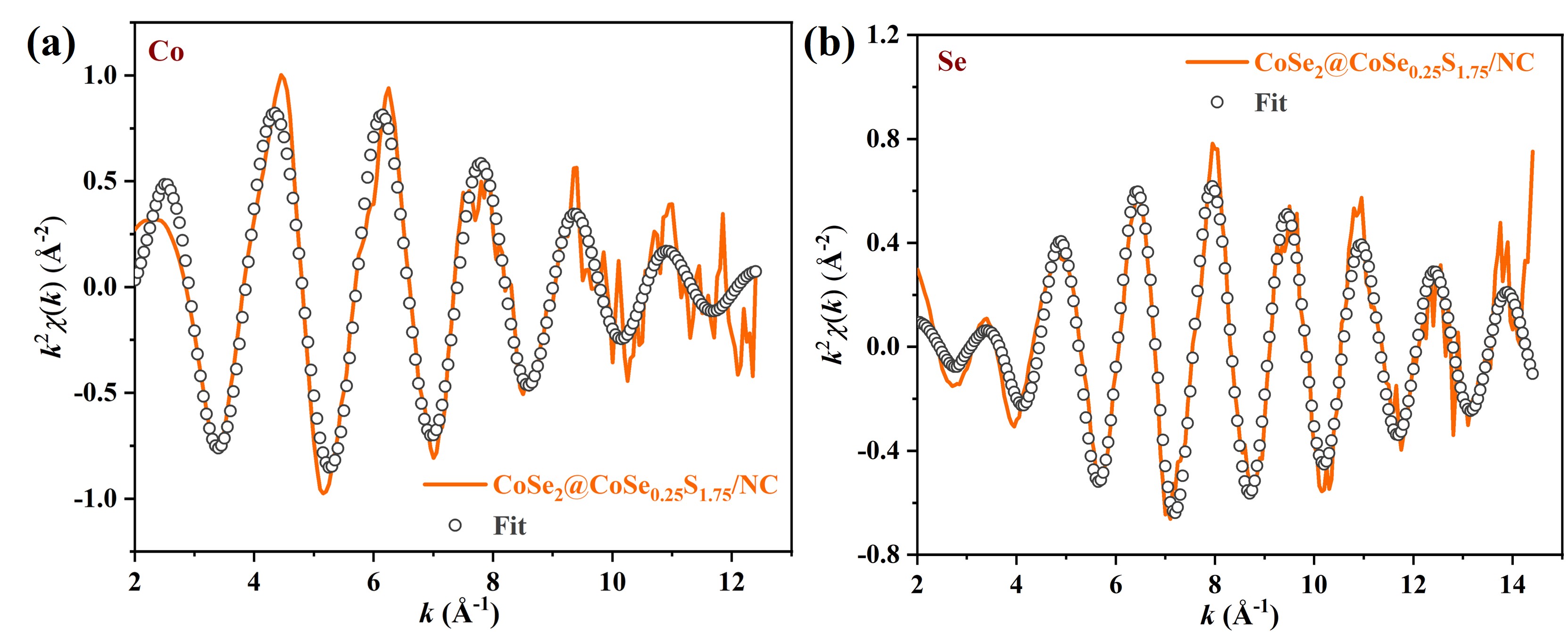


**Fig. S4** Fitted K3 space spectra of (a) Co and (b) Se in CoSe_2_@CoSe_0.25_S_1.75_/NC host.

**Table 1** EXAFS data fitting results of CoSe_2_@CoSe_0.25_S_1.75_/NC host.

| Sample | Path | *CN^a^* | *R*(Å)*^b^* | *σ*^2^ (Å^2^)*^c^* | Δ*E*_0_(eV)*^d^* | *R* factor |
| --- | --- | --- | --- | --- | --- | --- |
| Co K-edge (*Ѕ*_0_^2^=0.859) | | | | | | |
| Co foil | Co-Co | 12* | 2.489±0.001 | 0.0059 | 3.2 | 0.0013 |
| CoSe_2_@CoSe_0.25_S_1.75_/NC | Co-S | 3.8±0.2 | 2.290±0.012 | 0.0082 | 2.1 | 0.0092 |
|  | Co-Se | 1.2±0.3 | 2.403±0.018 |  | -7.3 |  |
| Se K-edge (*Ѕ*_0_^2^=0.883) | | | | | | |
| Se foil | Se-Se | 4* | 2.369±0.005 | 0.0050 | 5.2 | 0.0047 |
| CoSe_2_@CoSe_0.25_S_1.75_/NC | Se-S | 0.9±0.2 | 2.310±0.012 | 0.0041 | 6.3 | 0.0080 |
|  | Se-Co | 1.5±0.3 | 2.404±0.020 | 0.0032 | 5.5 |  |

*^a^CN*, coordination number; *^b^R*, the distance between absorber and backscatter atoms; *^c^σ*^2^, the Debye Waller factor value; *^d^ΔE*_0_, inner potential correction to account for the difference in the inner potential between the sample and the reference compound; *R* factor indicates the goodness of the fit. *S*_0_^2^ was fixed to 0.859 and 0.883, according to the experimental EXAFS fit of Co foil and Se foil by fixing *CN* as the known crystallographic value. * This value was fixed during EXAFS fitting, based on the known structure of Co and Se. Fitting conditions: *k* range: 3.0 - 11.5; *R* range: 1.0-3.0 (HG-23072301-Co); 3.0 - 13.0; *R* range: 1.0-3.0 (Sample-Se); Fitting space: R space; *k*-weight = 2. A reasonable range of EXAFS fitting parameters: 0.800 < *Ѕ*_0_^2^ < 1.000; *CN >* 0; *σ*^2^ > 0 Å^2^; |Δ*E*_0_| < 10 eV; *R* factor < 0.02.


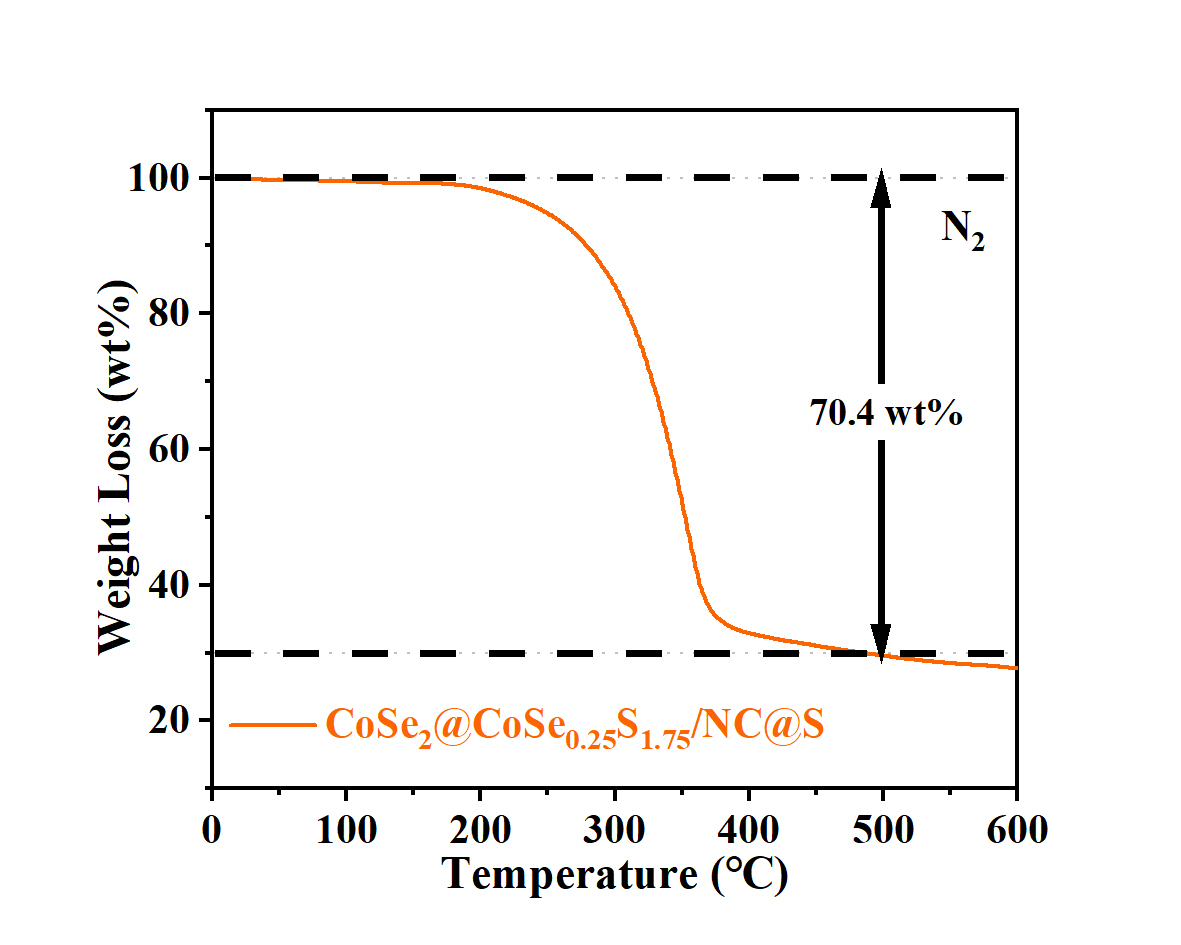


**Fig. S5** TG curve of CoSe_2_@CoSe_0.25_S_1.75_/NC@S cathode in N_2_.


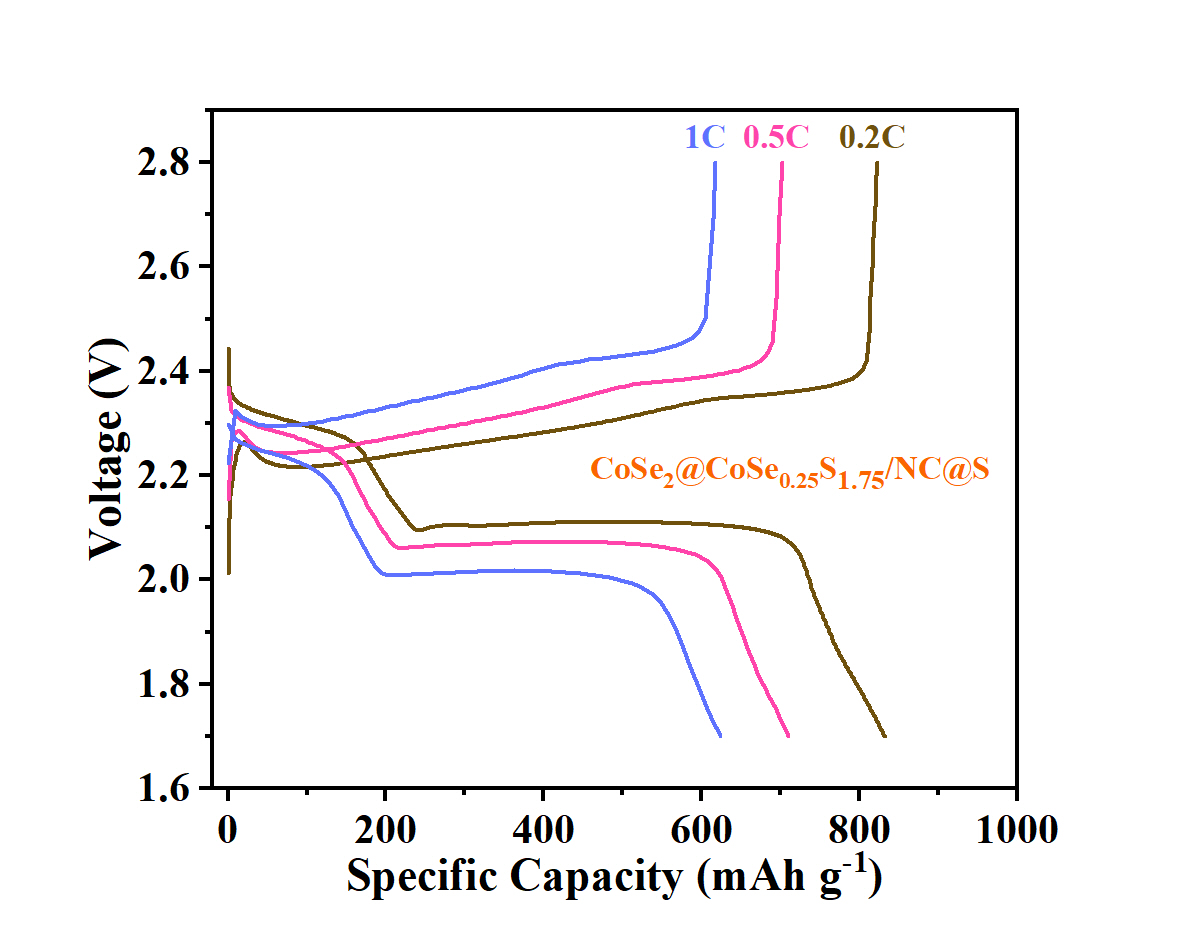


**Fig. S6** Charge and discharge profiles of CoSe_2_@CoSe_0.25_S_1.75_/NC@S cathode at different rates.


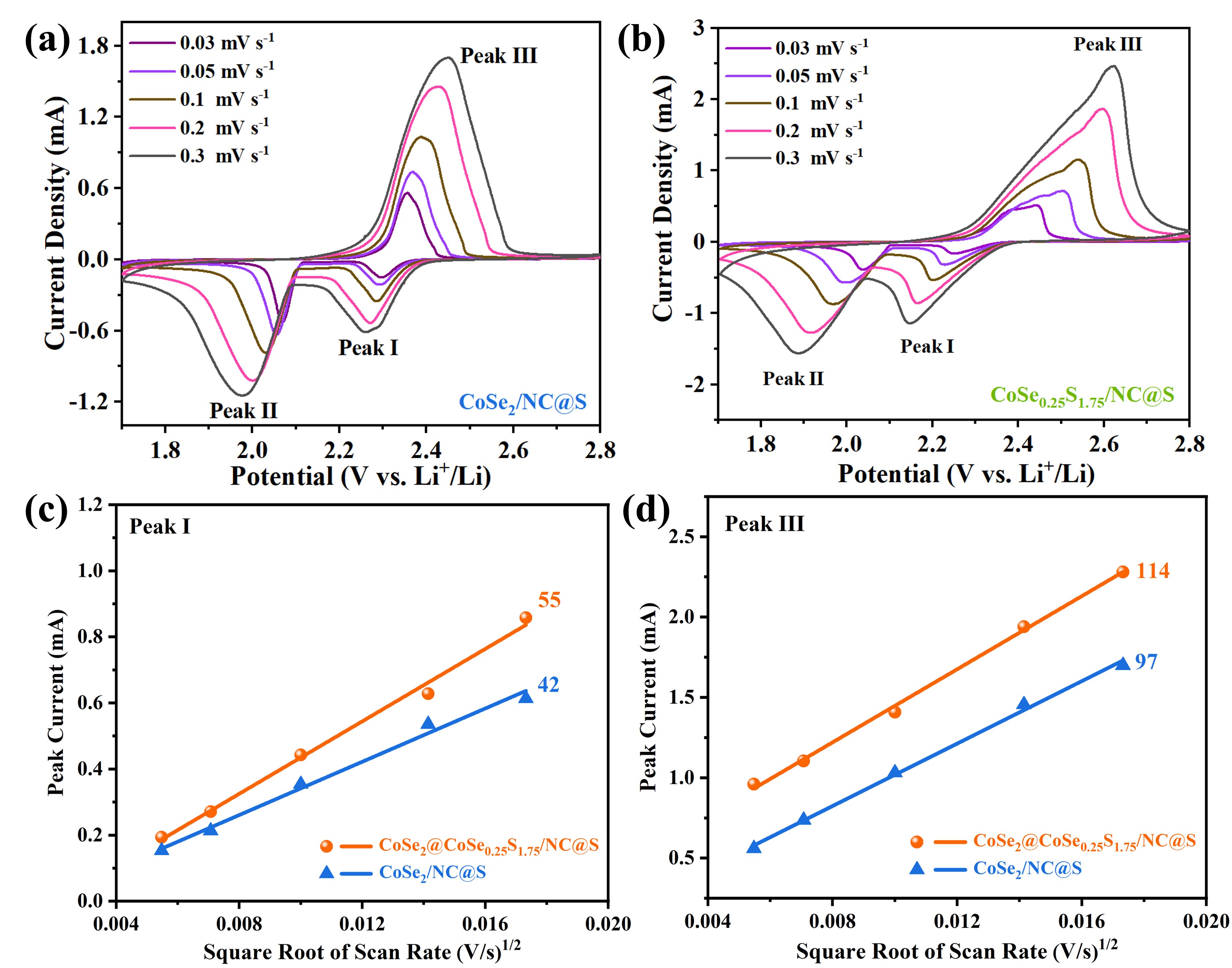


**Fig. S7** CV curves of (a) CoSe_2_/NC@S and (b) CoSe_2_@CoSe_0.25_S_1.75_/NC cathode at various scan rates. Linear fitting between currents of (c) Peak I and (d) Peak III and square root of scan rates for CoSe_2_@CoSe_0.25_S_1.75_/NC and CoSe_2_/NC@S cathode.


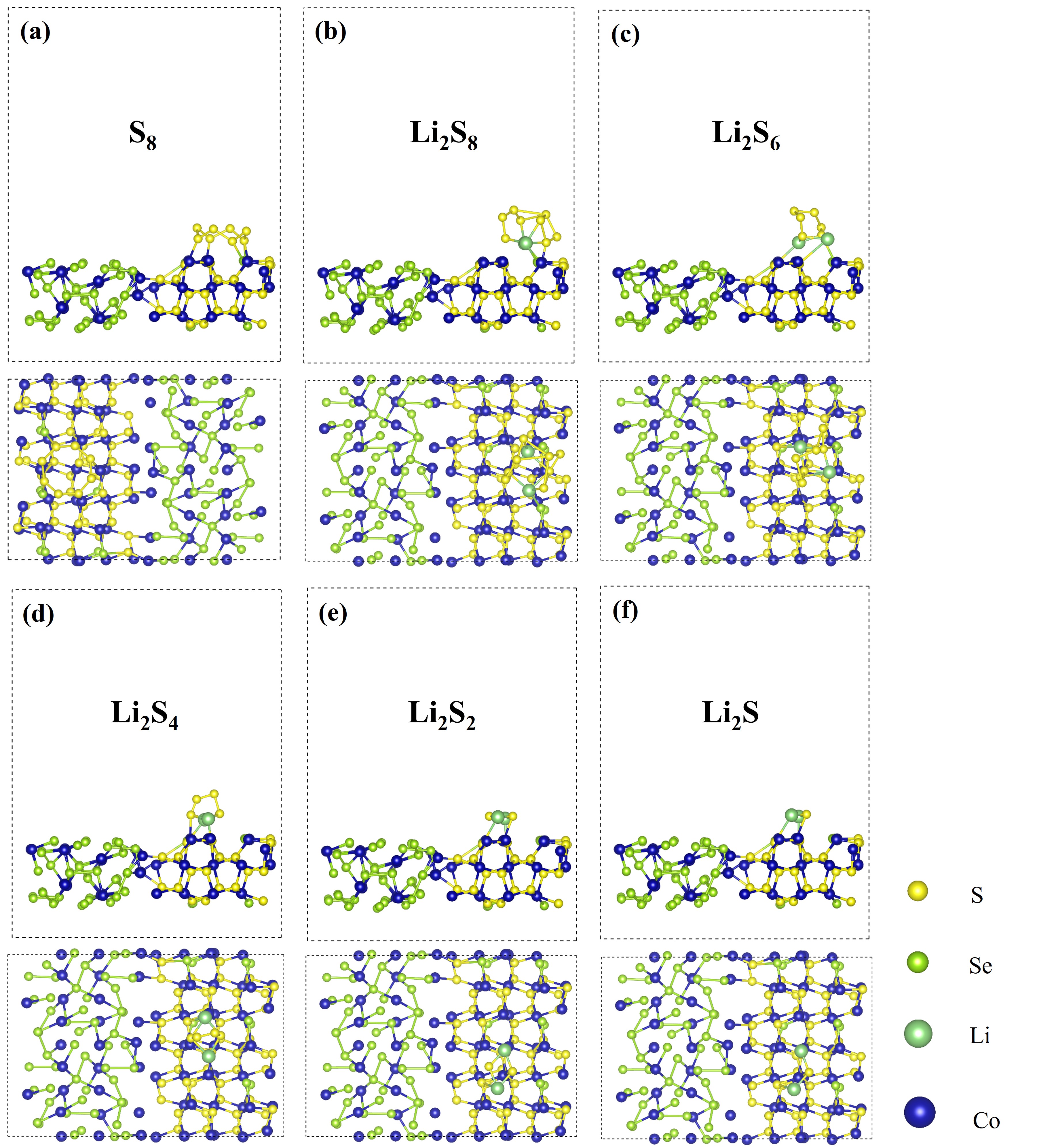


**Fig. S8** Optimized configurations of (a) S_8_, (b) Li_2_S_8_, (c) Li_2_S_6_, (d) Li_2_S_4_, (e) Li_2_S_2_ and (f) Li_2_S on A site (CoSe_0.25_S_0.75_) of CoSe_2_@CoSe_0.25_S_0.75_ heterostructure (Upper: side view, Lower: top view).


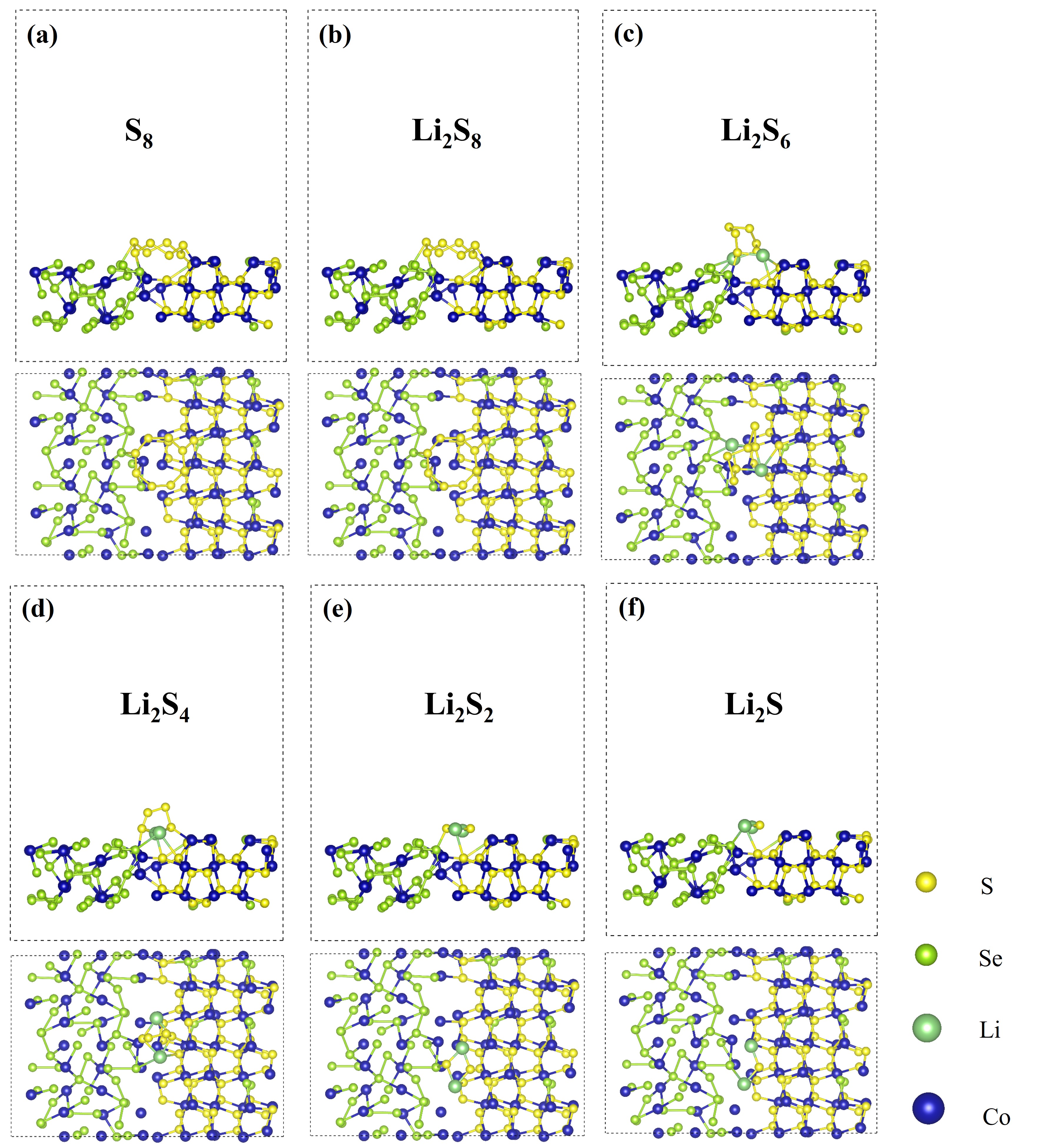


**Fig. S9** Optimized configurations of (a) S_8_, (b) Li_2_S_8_, (c) Li_2_S_6_, (d) Li_2_S_4_, (e) Li_2_S_2_ and (f) Li_2_S on B site (heterointerface) of CoSe_2_@CoSe_0.25_S_0.75_ heterostructure (Upper: side view, Lower: top view).


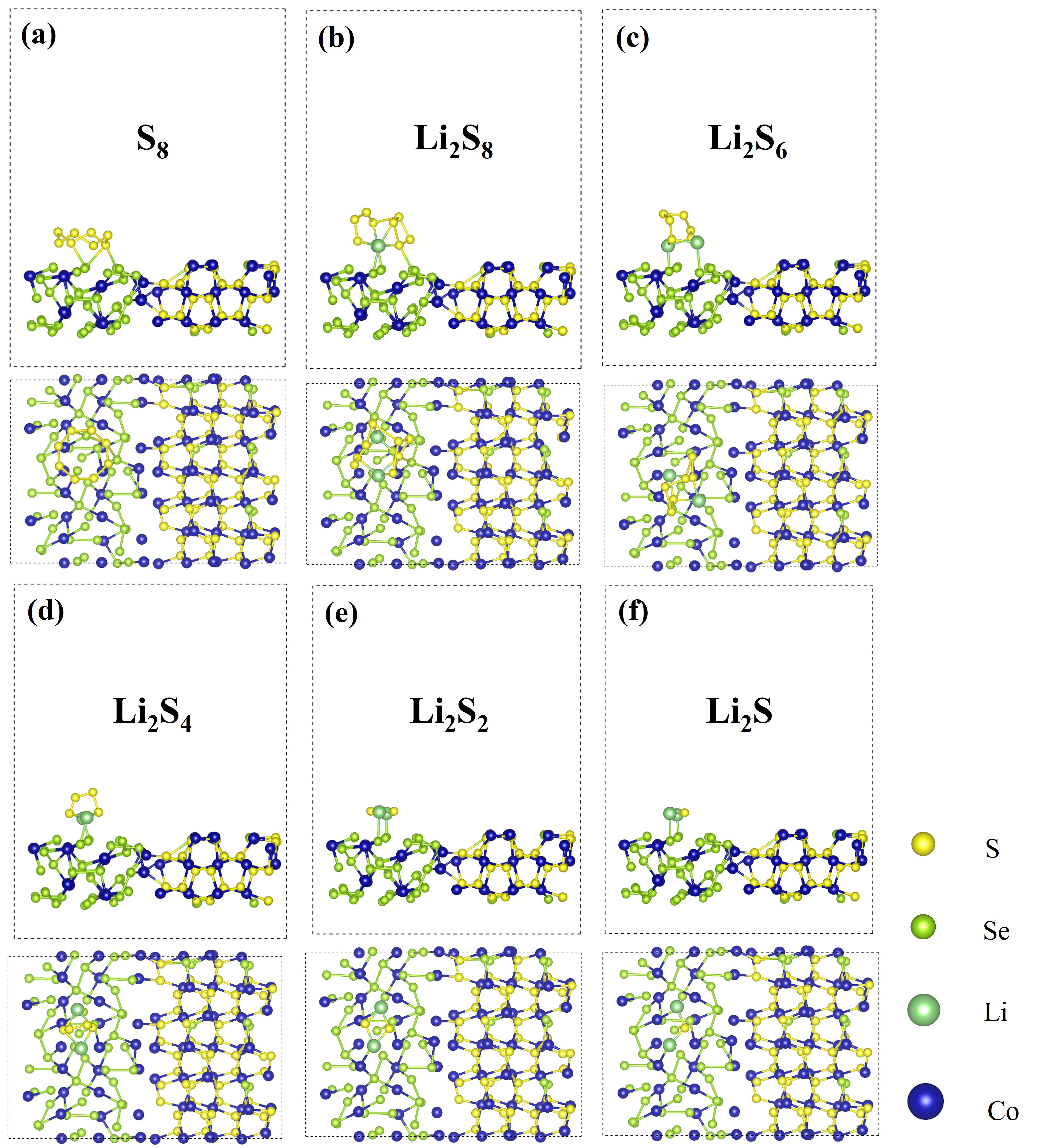


**Fig. S10** Optimized configurations of (a) S_8_, (b) Li_2_S_8_, (c) Li_2_S_6_, (d) Li_2_S_4_, (e) Li_2_S_2_ and (f) Li_2_S on C site (CoSe_2_) of CoSe_2_@CoSe_0.25_S_0.75_ heterostructure (Upper: side view, Lower: top view).


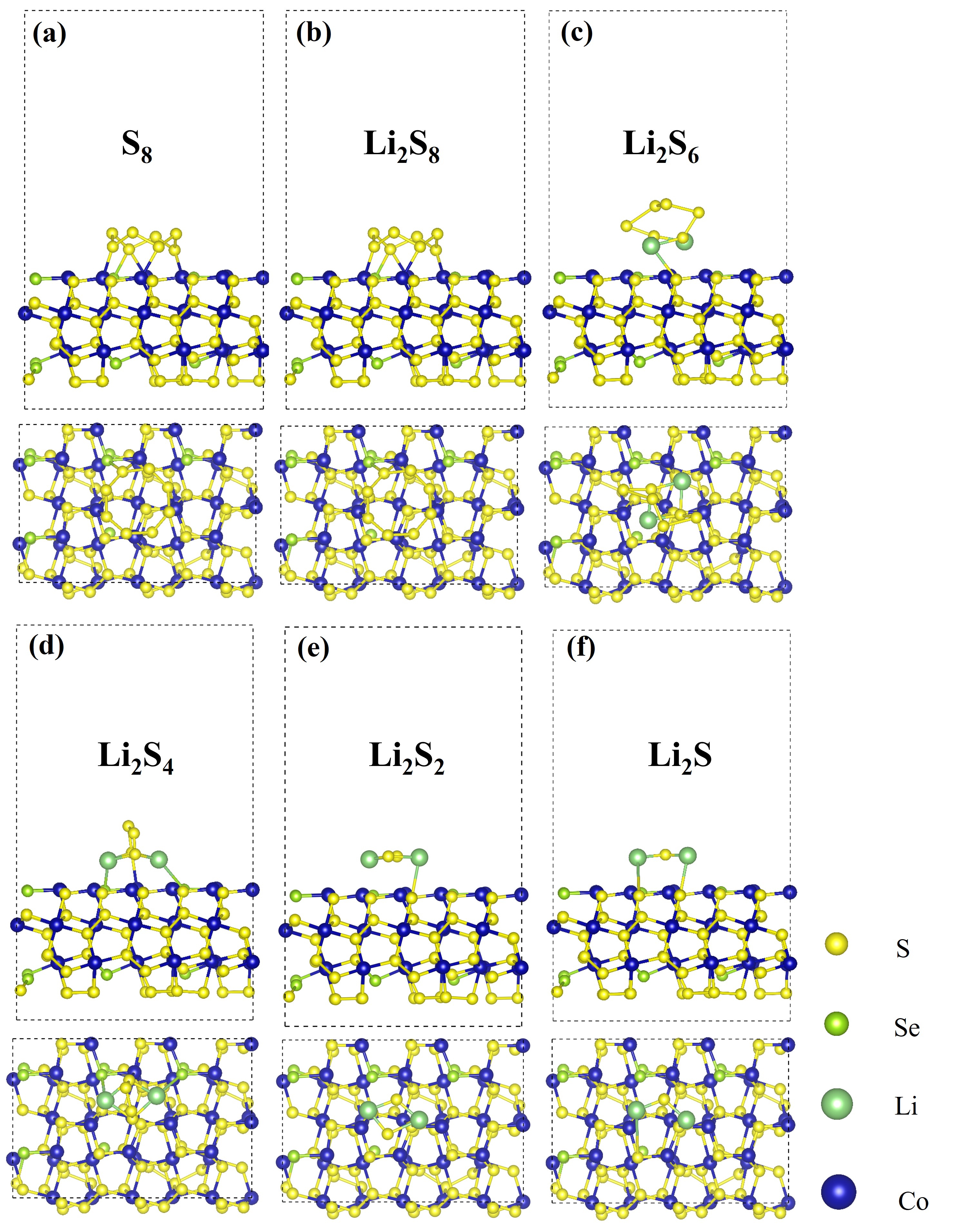


**Fig. S11** Optimized configurations of (a) S_8_, (b) Li_2_S_8_, (c) Li_2_S_6_, (d) Li_2_S_4_, (e) Li_2_S_2_ and (f) Li_2_S on CoSe_0.25_S_0.75_ (Upper: side view, Lower: top view).


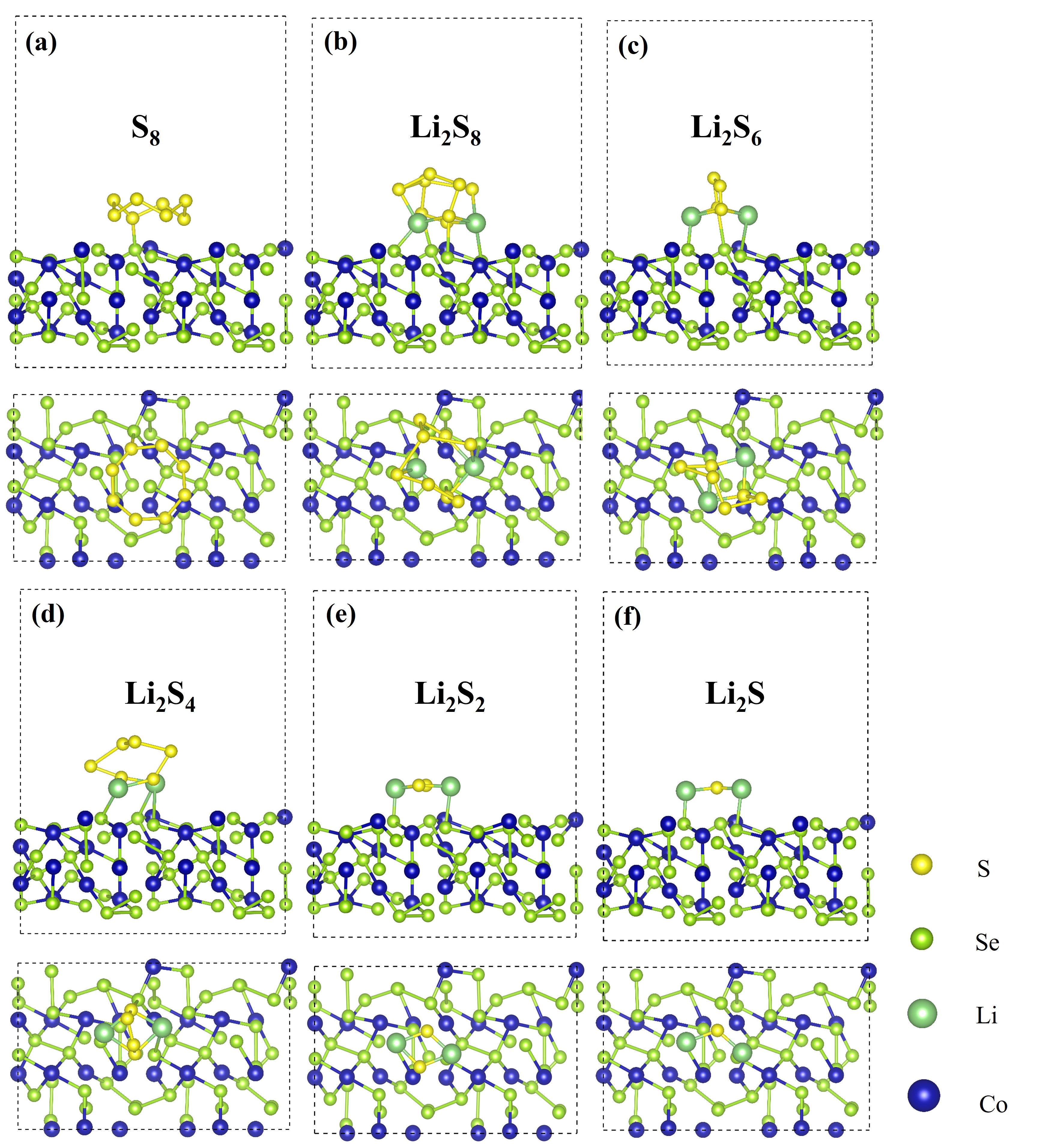


**Fig. S12** Optimized configurations of (a) S_8_, (b) Li_2_S_8_, (c) Li_2_S_6_, (d) Li_2_S_4_, (e) Li_2_S_2_ and (f) Li_2_S on CoSe_2_ (Upper: side view, Lower: top view).


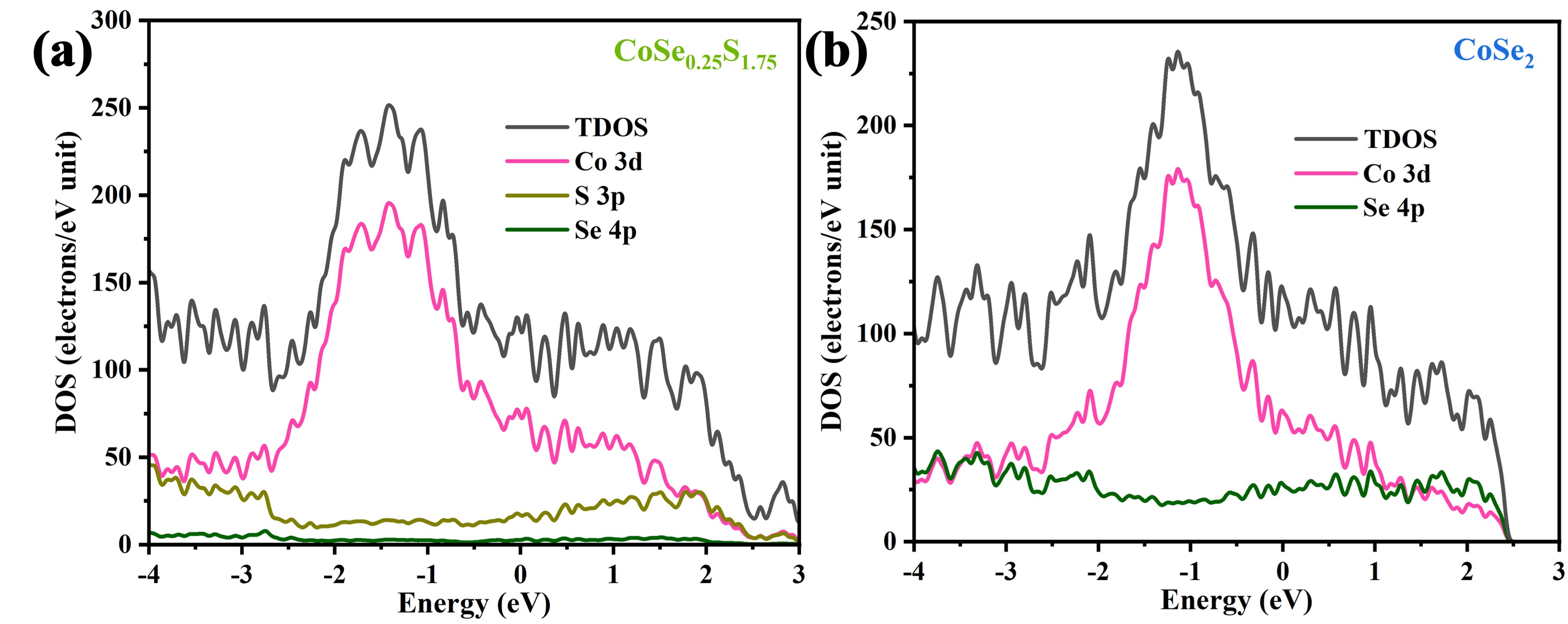


**Fig. S13** (a) DOS of Co 3d, S 3p and Se 4p and TDOS in CoSe_0.25_S_0.75_. (b) DOS of Co 3d and Se 4p and TDOS in CoSe_2_.


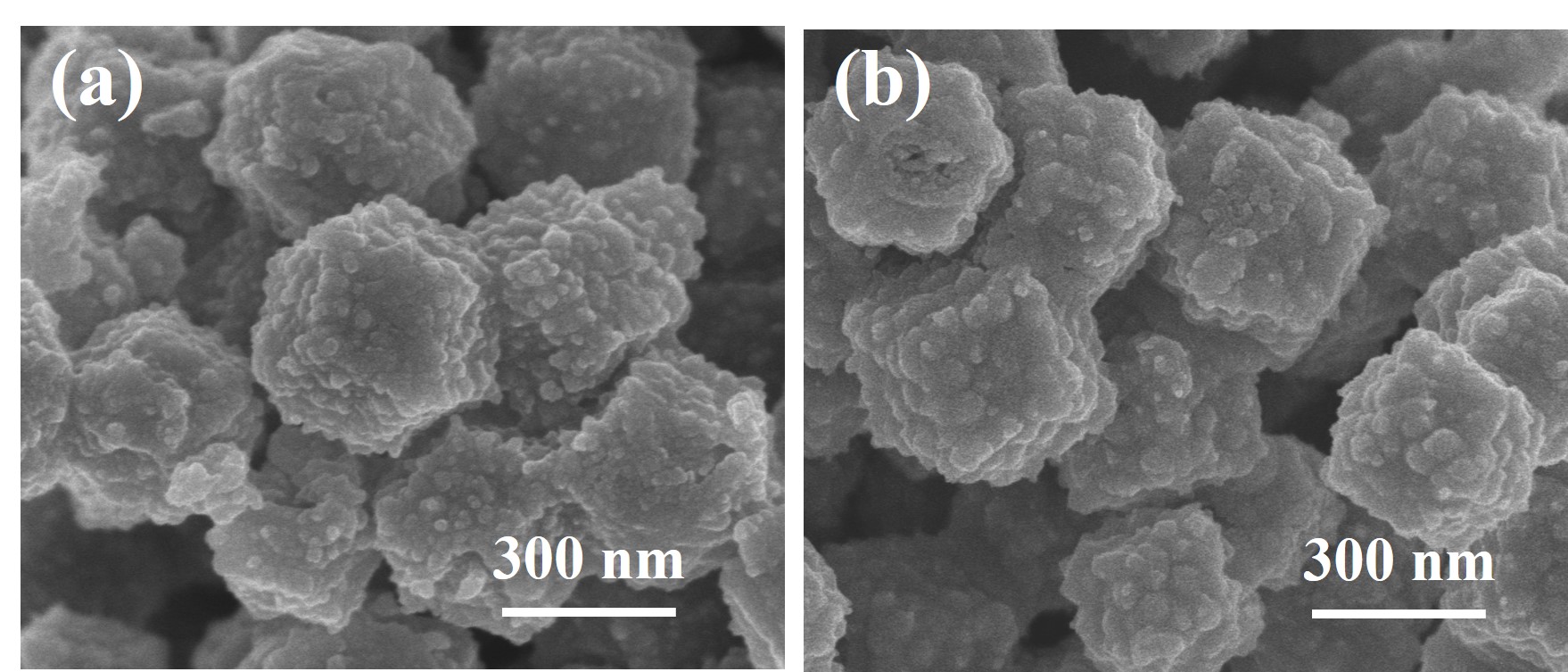


**Fig. S14** SEM image of (a) CoSe_2_@CoS_2_/NC and (b) CoS_2_/NC host.


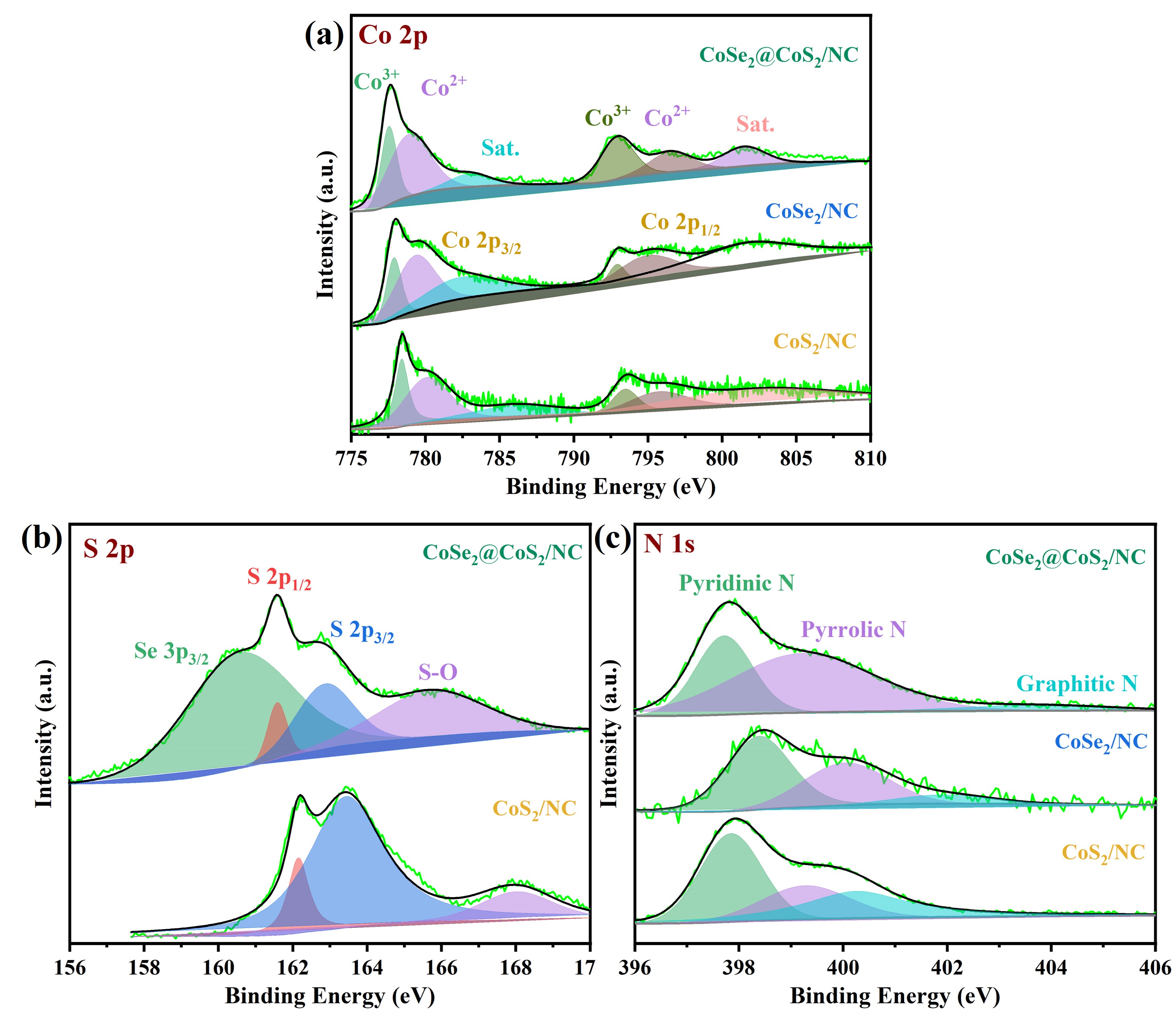


**Fig. S15** High-resolution (a) Co 2p and (c) N 1s XPS spectra of CoSe_2_@CoS_2_/NC, CoSe_2_/NC and CoS_2_/NC host. (b) High-resolution S 2p XPS spectra of CoSe_2_@CoS_2_/NC and CoS_2_/NC host.


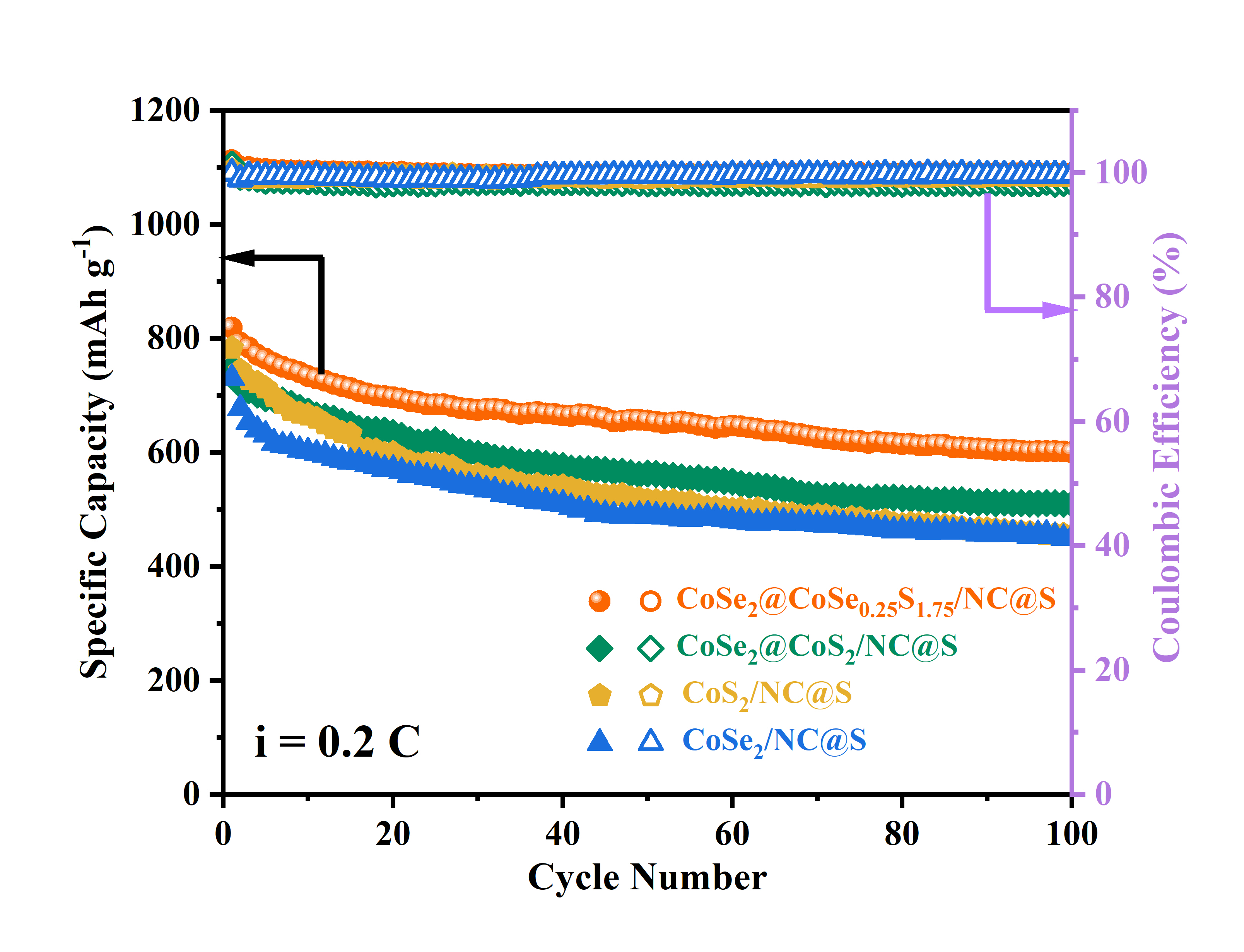


**Fig. S16** Cycling performances of CoSe_2_@CoSe_0.25_S_1.75_/NC@S, CoSe_2_@CoS_2_/NC@S, CoSe_2_/NC@S and CoS_2_/NC@S cathode at 0.2 C.


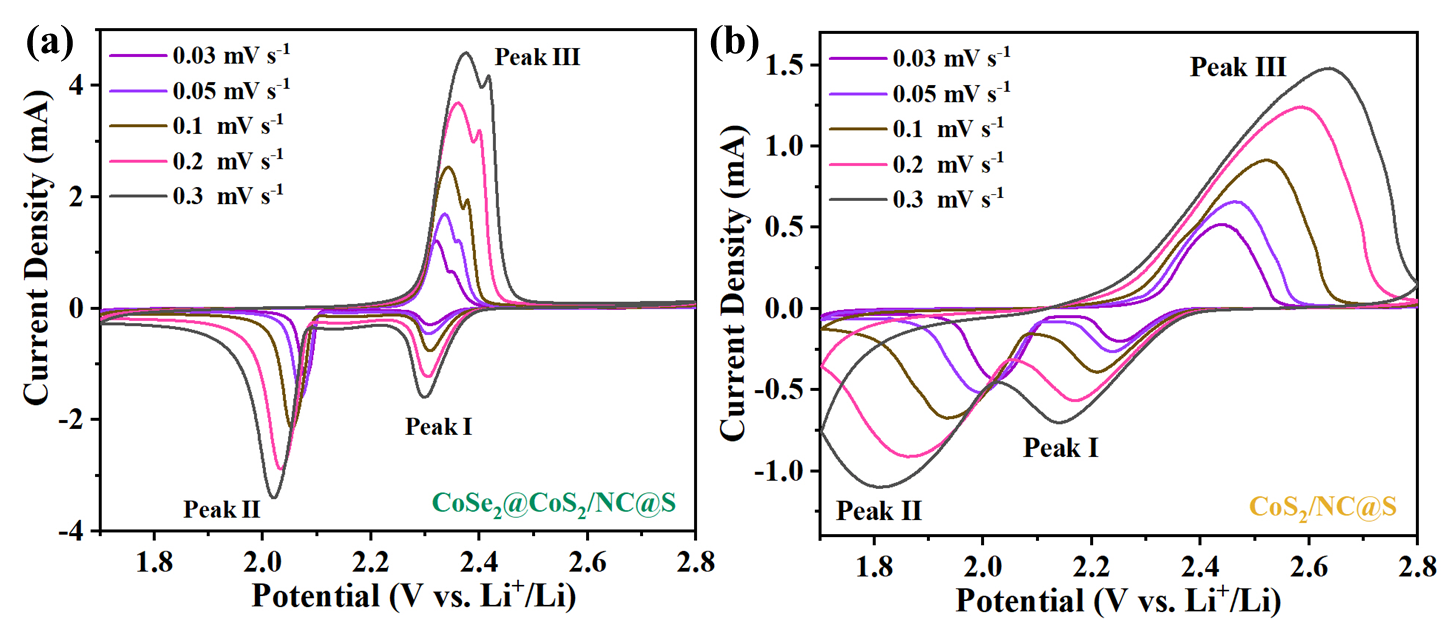


**Fig. S17** CV curves of (a) CoSe_2_@CoS_2_/NC@S and (b) CoS_2_/NC@S cathode at various scan rates.


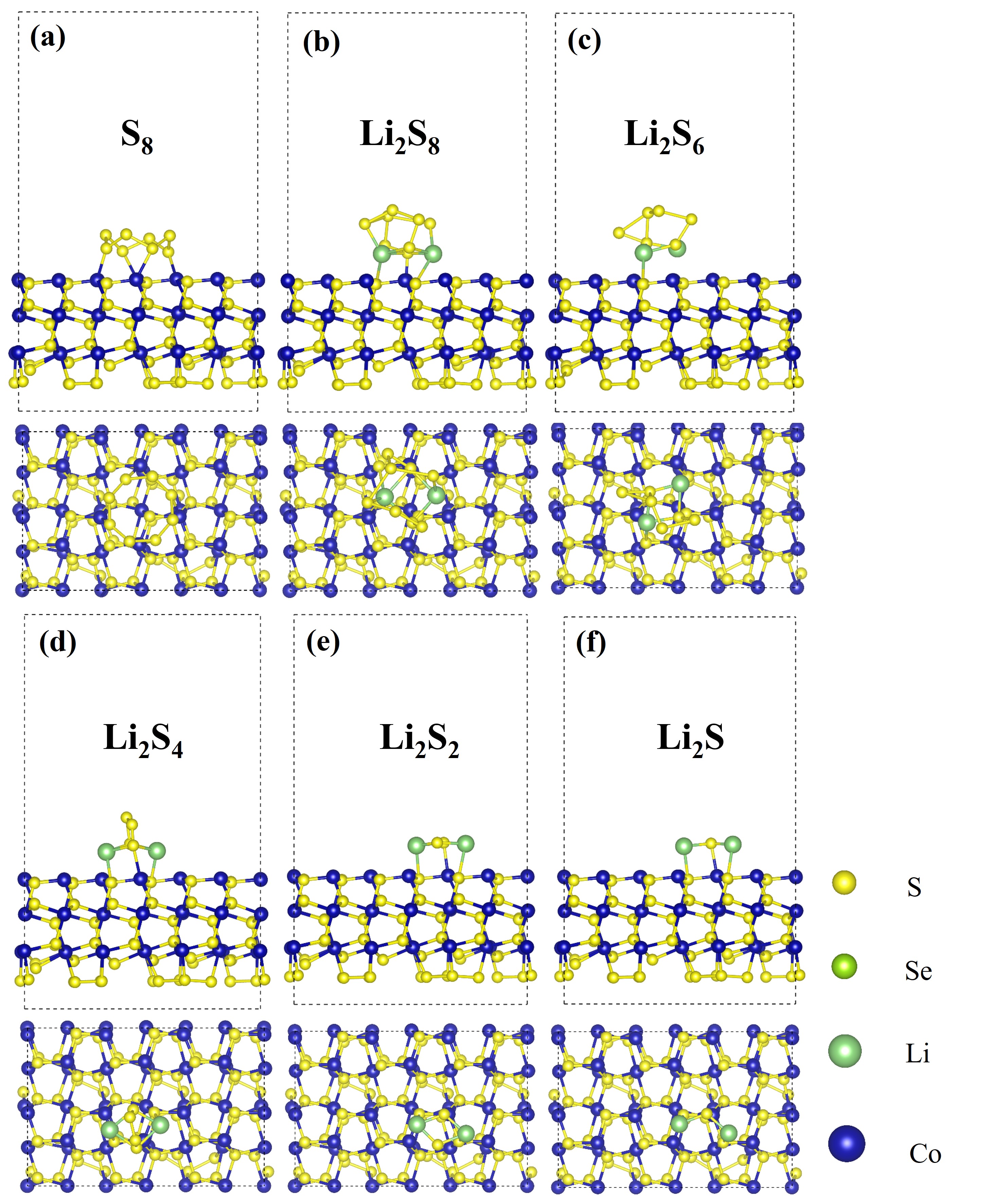


**Fig. S18** Optimized configurations of (a) S_8_, (b) Li_2_S_8_, (c) Li_2_S_6_, (d) Li_2_S_4_, (e) Li_2_S_2_ and (f) Li_2_S on CoS_2_ (Upper: side view, Lower: top view).


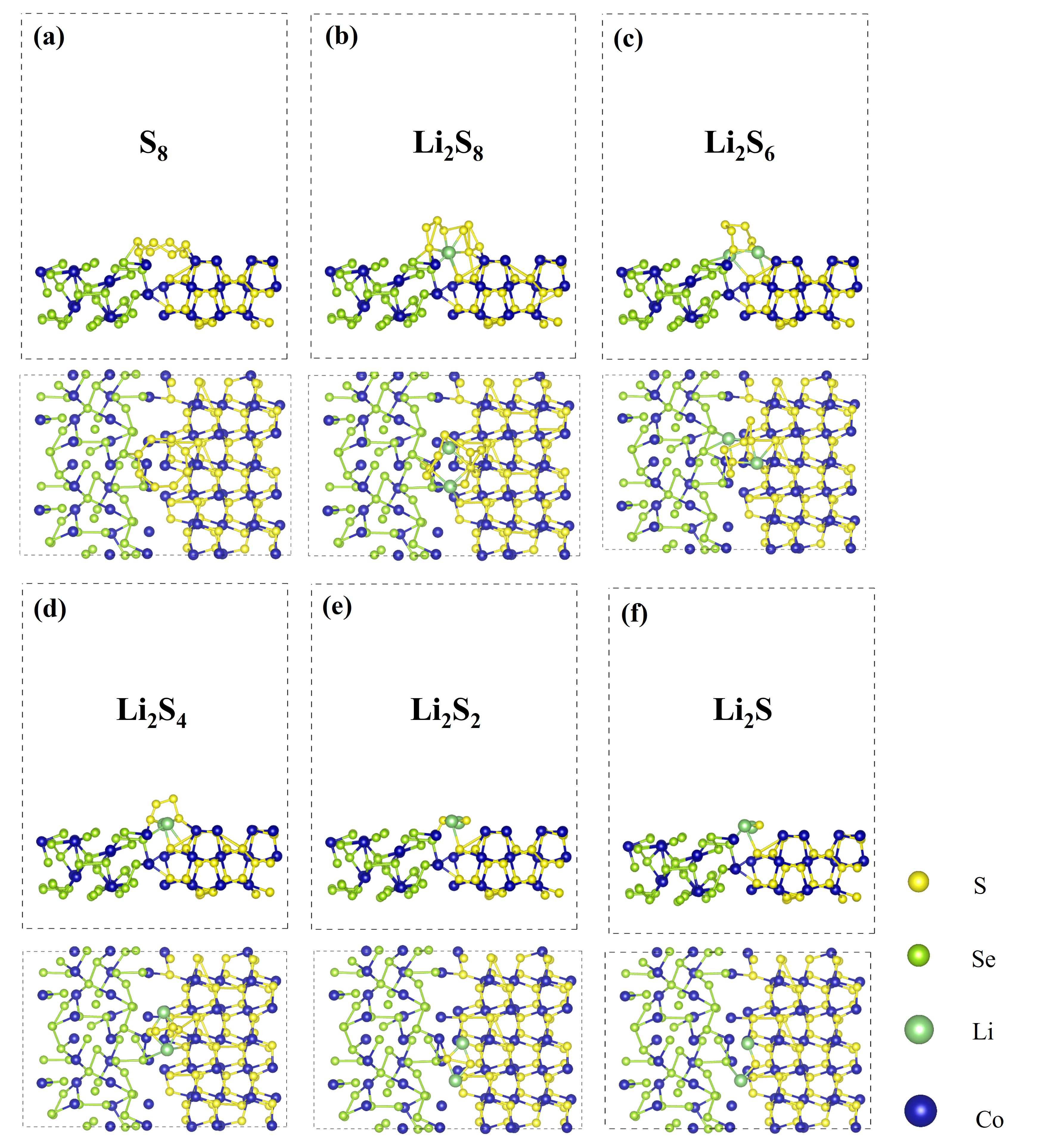


**Fig. S19** Optimized configurations of (a) S_8_, (b) Li_2_S_8_, (c) Li_2_S_6_, (d) Li_2_S_4_, (e) Li_2_S_2_ and (f) Li_2_S on CoSe_2_@CoS_2_ (Upper: side view, Lower: top view).


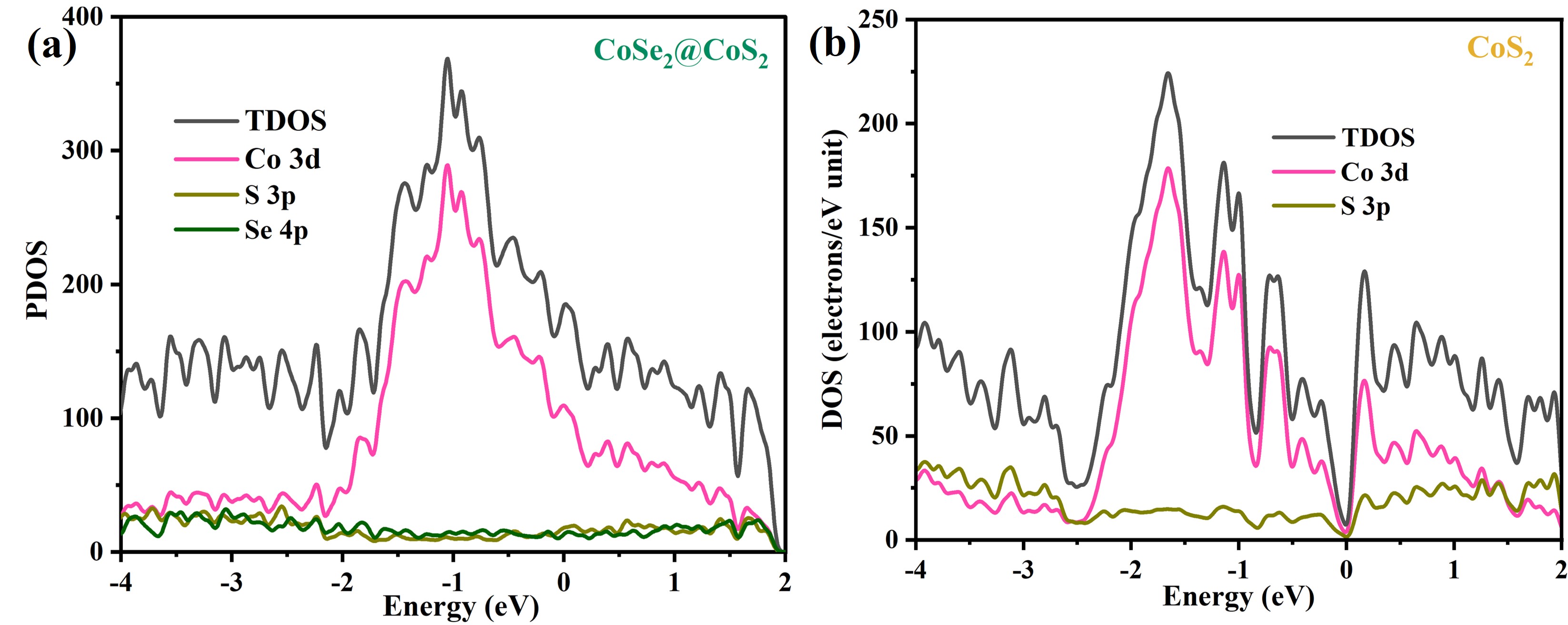


**Fig. S20** (a) PDOS of Co 3d, S 3p and Se 4p and TDOS in CoSe_2_@CoS_2_. (b) DOS of Co 3d and S 3p and TDOS in CoS_2_.


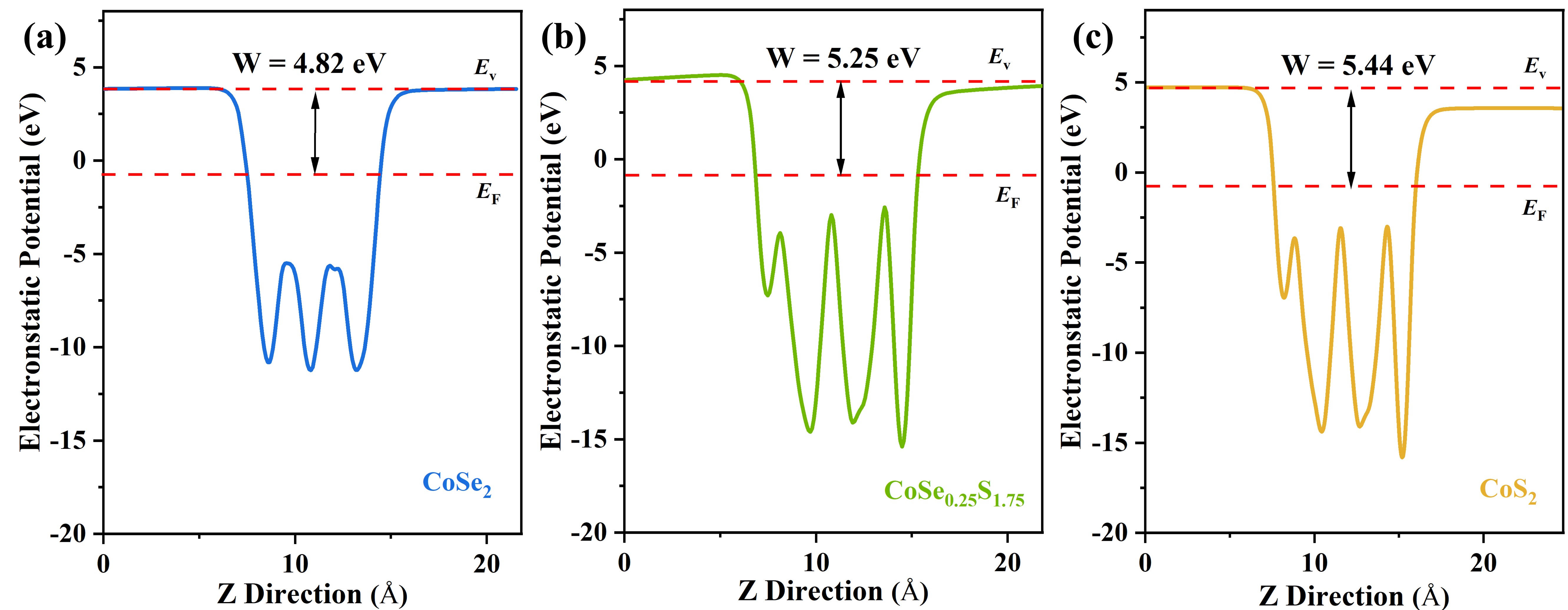


**Fig. S21** Work function of (a) CoSe_2_, (b) CoSe_0.25_S_0.75_ and (c) CoS_2._ (*E*_v_: Vacuum level, *E*_f_ : Fermi level).

.

**
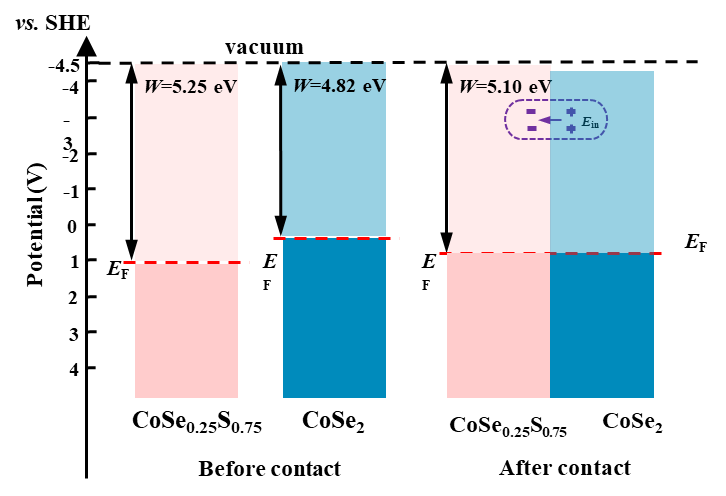
**

**Fig. S22** Schematic diagram of the formation process of the built-in electric field at CoSe_2_@CoSe_0.25_S_0.75_ heterointerface.


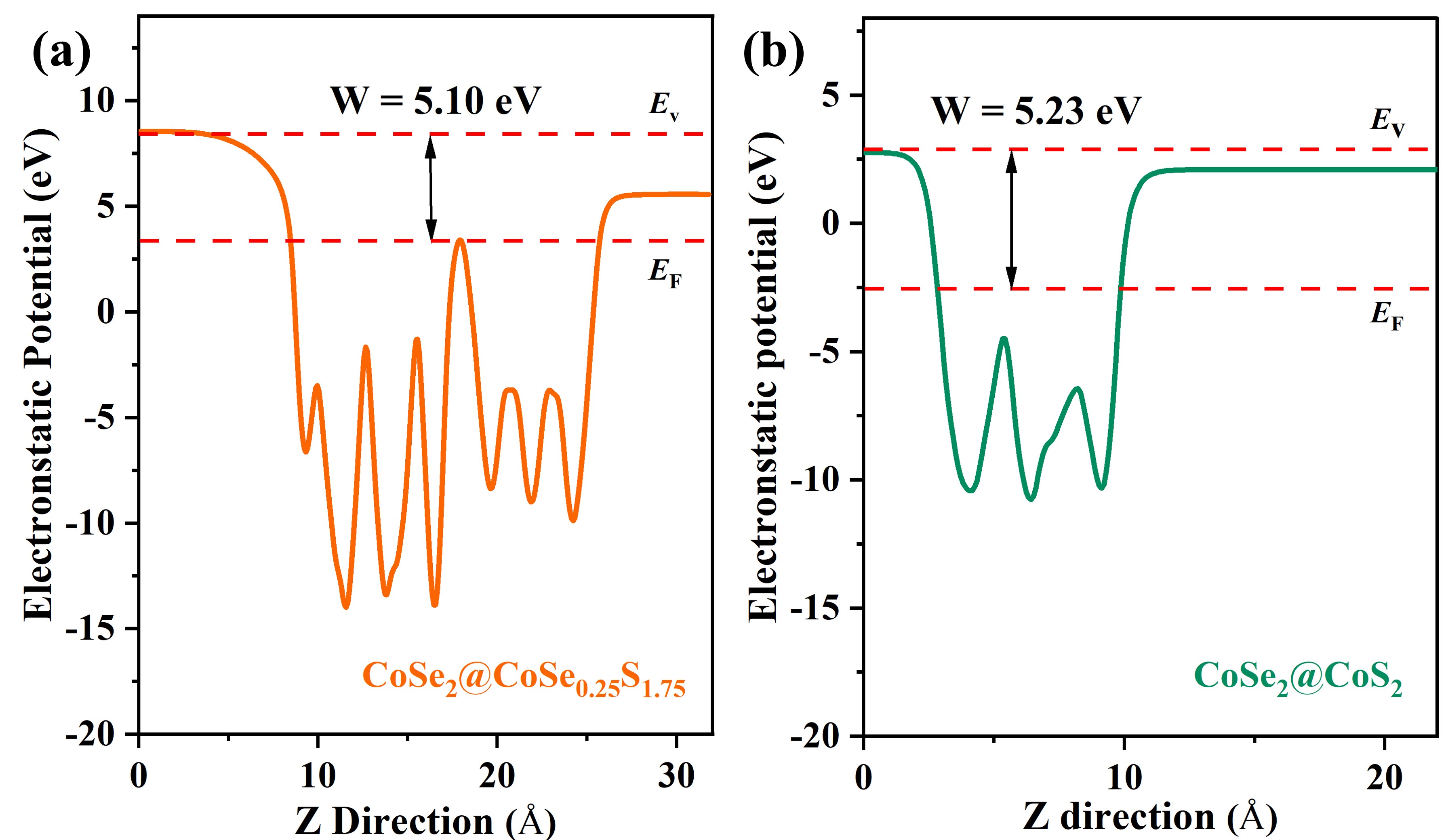


**Fig. S23** Work function of (a) CoSe_2_@CoSe_0.25_S_0.75_ and (b) CoSe_2_@CoS_2._ (*E*_v_: Vacuum level, *E*_f_ : Fermi level).


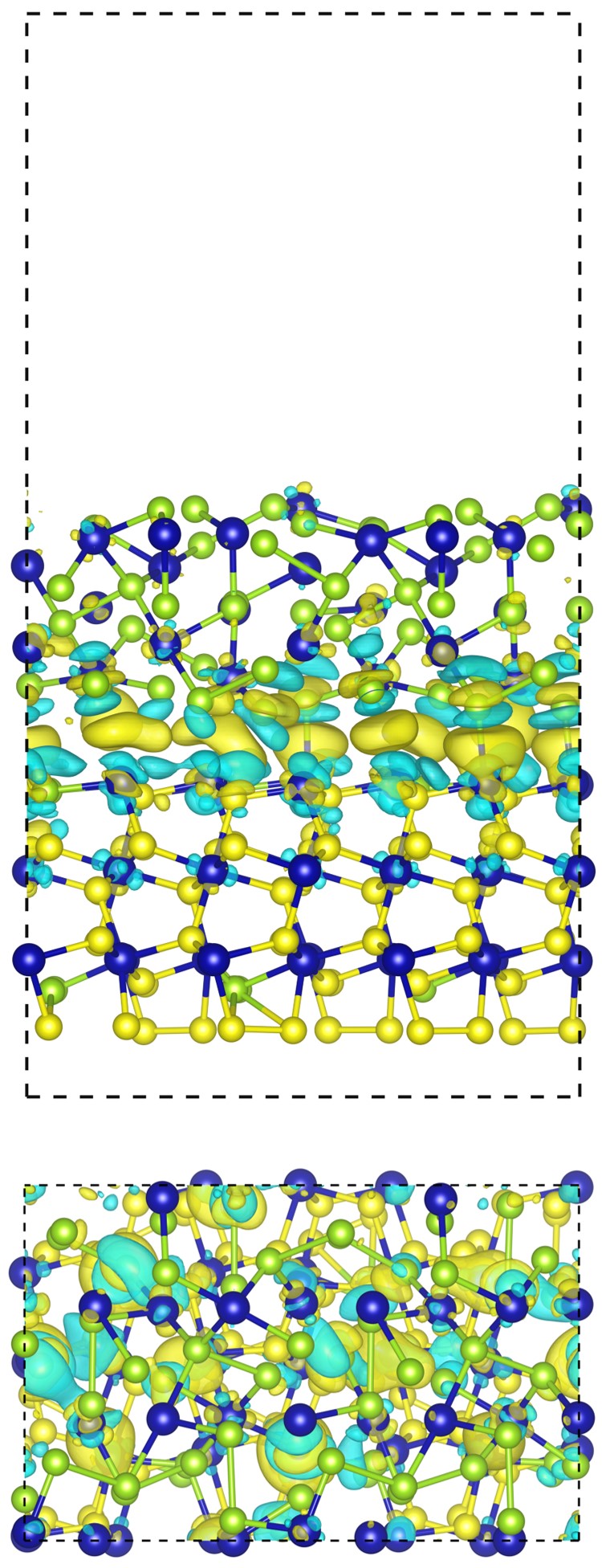


**Fig. S24** Differential charge density diagram of CoSe_2_@CoSe_0.25_S_0.75_ heterostructure. (Upper: side view, Lower: top view, Green and yellow represent electron depletion and electron accumulation with the isosurface value of 0.0015 e/Bohr^3^, respectively.)

# References

[1] S. Yang, L. Peng, P. Huang, X. Wang, Y. Sun, C. Cao, W. Song, *Angew. Chem. Int. Ed. Engl.* **2016**, *55*, 4016-4020.

[2] aS. I. Zabinsky, J. J. Rehr, A. Ankudinov, R. C. Albers, M. J. Eller, *Phys. Rev. B* **1995**, *52*, 2995-3009; bB. Ravel, M. Newville, *J. Synchrotron Radiat.* **2005**, *12*, 537-541.

[3] G. Kresse, D. Joubert, *Phys. Rev. B* **1999**, *59*, 1758-1777.

[4] J. P. Perdew, K. Burke, M. Ernzerhof, *Phys. Rev. Lett.* **1996**, *77*, 3865-3868.

[5] H. Zhang, H. Cui, J. Li, Y. Liu, Y. Yang, M. Wang, *Nanoscale* **2019**, *11*, 21532-21541.
